# Phenotypic and genomic characterization of a halophilic sulfate-reducing bacterium *Desulfoplanes aerotolerans* sp. nov. from a petroleum reservoir and its possible implication in pipeline corrosion

**DOI:** 10.3389/fmicb.2026.1968038

**Published:** 2026-10-14

**Authors:** Salimat K. Bidzhieva, Tatiana P. Tourova, Denis S. Grouzdev, Salima R. Soloveva, Diyana S. Sokolova, Vera M. Tereshina, Andrey B. Poltaraus, Andrey V. Mardanov, Tamara N. Nazina

**Affiliations:** 1 Winogradsky Institute of Microbiology, Research Center of Biotechnology, Russian Academy of Sciences, Moscow, Russia; 2 School of Marine and Atmospheric Sciences, Stony Brook University, Stony Brook, NY, United States; 3 Engelhardt Institute of Molecular Biology, Russian Academy of Sciences, Moscow, Russia; 4 Institute of Bioengineering, Research Center of Biotechnology, Russian Academy of Sciences, Moscow, Russia

**Keywords:** *Desulfoplanes*, genomic analysis, microbiologically induced corrosion, petroleum reservoir, polyphasic taxonomy, sulfate-reducing bacteria

## Abstract

**Introduction:**

The issue of biogenic corrosion of steel equipment is especially pressing for petroleum reservoirs with high-salinity formation water containing sulfate. The predominance of sulfate-reducing bacteria of the genus *Desulfoplanes* in formation water samples from petroleum reservoirs in Tatarstan (Russia) was previously demonstrated using 16S rRNA gene amplicon sequencing and metagenomic analysis. The present study is devoted to the isolation, phenotypic and genomic characterization of the sulfate-reducing bacterial strain *Desulfoplanes* sp. PS50^T^ and its involvement in steel corrosion. Studying the composition of sulfidogenic prokaryotes and their corrosion activity is necessary in order to find a way to suppress their growth and minimize corrosion of oilfield equipment.

**Methods:**

A new sulfate-reducing bacterial strain *Desulfoplanes* sp. PS50^T^ was studied using polyphase taxonomy approaches, including genome sequencing and functional annotation. The ability to cause corrosion of the steel coupon and the effect of three biocides on the growth of the strain were tested.

**Results:**

Sulfate-reducing strain *Desulfoplanes* sp. PS50^T^ isolated from an Arkhangelskoe petroleum reservoir (Tatarstan, Russia) was phenotypically characterized. The optimal growth of the strain PS50^T^ was observed at a temperature of 25–30 °C, in the presence of 40–50 g NaCl·L^−1^ and at pH 7.5. The strain utilized formate, lactate, yeast extract, and H_2_/CO_2_/acetate as carbon and energy sources for reduction of sulfate to sulfide. The strain exhibited both anaerobic and microaerophilic growth (in the presence of 5% O_2_ (*v/v*). Based on physiological features and genomic and phylogenomic analyses, strain PS50^T^ (=VKM B-4014^T^ = KCTC 35101^T^) has been assigned to a new species, *Desulfoplanes aerotolerans* sp. nov. Genomic analysis revealed genes of sulfate reduction, hydrogenase, and iron uptake, which confirms the strain’s ability to participate in the steel biocorrosion. The genome also contains the genes that determine the main metabolic pathways and the strain’s adaptation to the high-salinity conditions of its habitat.

**Discussion:**

This study provides experimental proofs on the ability of sulfate-reducing bacteria of the genus *Desulfoplanes* to participate in general and pitting corrosion, confirmed by deep genome-resolved detection of corrosion-determining genes.

## Introduction

Since the pioneering works of Bastin and Ginzburg-Karagicheva, carried out in 1926, the problem of corrosion in the oil industry has been associated with the activity of sulfate-reducing bacteria (Bastin, 1926; Ginzburg-Karagicheva, 1926; Magot et al., 2000; Liu et al., 2019). Microbiologically influenced corrosion (MIC) and oil souring are the main problems that arise when using water-flooding methods that lead to activation of the subsurface microbial community, as well as when using microbiological methods of enhancement of oil recovery (MEOR) (Nazina et al., 2017; Youssef et al., 2009; Gieg et al., 2010). One of the ways to suppress sulfate-reducing bacteria (SRB) growth is to inject biocides into the reservoir, which selectively inhibit SRB growth and decrease sulfide formation in reservoir water (Gieg et al., 2011; Senthilmurugan et al., 2019; Xu et al., 2023). However, the formation of microbial biofilms in the formation decreases the sensitivity of sulfidogens to the applied biocides (Vigneron et al., 2016; Elumalai et al., 2021; Shi et al., 2021; Pereira et al., 2021). It is necessary to study the composition of corrosive prokaryotes and pure cultures of sulfidogens in order to find the approach to suppress their growth and minimize corrosion of oilfield equipment.

In recent decades, significant progress has been made in the study of corrosion-active prokaryotes and mechanisms of biogenic corrosion of steel equipment (Davidova et al., 2022; Knisz et al., 2023; Thakur et al., 2023). It has been shown that, along with sulfate-reducing bacteria (SRB) and archaea (SRA), acid-forming anaerobic fermentative bacteria that produce volatile acids and carbonic acid, and are capable of producing sulfide by reducing oxidized sulfur compounds other than sulfate (sulfite, thiosulfate, and elemental sulfur) also cause corrosion (Magot et al., 1997; Duncan et al., 2009; Sokolova et al., 2021). The chemical interaction of sulfide/hydrogen sulfide with steel equipment leads to general corrosion of steel. Sulfide production is also accompanied by a decrease in rock permeability, deterioration of oil quality, and environmental problems during oil extraction, transportation, and refining (Ibrahim et al., 2018; Gao and Fan, 2023; Grigoryan et al., 2025).

Moreover, it has been shown that, apart from heterotrophic sulfidogenic bacteria and archaea autotrophic SRBs, as well as methanogens also participate in metal corrosion. The latter reduce carbon dioxide/carbonates from reservoir water to methane using electrons directly from iron, which leads to pitting corrosion (Daniels et al., 1987; Uchiyama et al., 2010; Venzlaff et al., 2013; Liang et al., 2014, 2016). In addition to methanogens, pitting corrosion can be caused by other hydrogen-using prokaryotes, for example, obligate chemolithotrophic acetogens capable of growing on H_2_/CO_2_ to form acetate, and in the absence of molecular hydrogen receiving electrons from ferrous iron (Fe^2+^) (Mori et al., 2010; Kato et al., 2015; Mand et al., 2016; Tang et al., 2021). The simultaneous presence of autotrophic and heterotrophic SRBs, autotrophic acetogens and methanogens has been shown at the Prirazlomnoye oil field, where they are probably the cause of corrosion of steel equipment (Sokolova et al., 2025).

A number of Tatarstan oil fields contain high-salinity reservoir waters with a high level of sulfates. Oil recovery at such sites is complicated by biogenic corrosion of steel equipment. To determine the agents of microbial corrosion and to carry out the subsequent search for effective biocides, it is necessary to isolate and study corrosion-active prokaryotes.

This work is part of a study of prokaryotes that induce corrosion of steel equipment in the oil reservoirs of Tatarstan (Russia) (Nazina et al., 2020; Ziganshina et al., 2023; Bidzhieva et al., 2024a). One of the objects of research was the Arkhangelskoe oil field with carbonate reservoirs, highly mineralized reservoir water, and freshwater flooding. Samples of production and injection water from the Arkhangelskoe field have been repeatedly studied using culture, metagenomic, and radioisotope methods (Nazina et al., 2017; Kadnikov et al., 2023). In water samples collected in 2016, the total salinity of reservoir water in the zone of mixing with fresh injected water decreased from 244.1 to 36.2 g·L^−1^, which led to an increase in the rate of sulfate reduction in the studied samples from zero values to 750 and 2,610 ng·S^2−^·L^−1^·day^−1^, while methanogenesis from acetate increased to 1722 nL CH_4_·L^−1^·day^−1^ (Nazina et al., 2020). By 16S rRNA gene V3 − V4 regions profiling in production water samples were revealed aerobic organotrophic bacteria of the genera *Bacillus*, *Pseudomonas*, and *Methylobacterium*, anaerobic fermentative bacteria of the genera *Halanaerobium*, *Geotoga*, and *Mesotoga*, syntrophic bacteria *Smithella* and *Syntrophus*, sulfate-reducing bacteria of the genera *Desulfocurvus*, *Desulfonatronovibrio*, and *Desulfovibrio*, and halophilic methanogens of the genus *Methanohalophilus* (PRJNA589080).

In 2021, using amplicon sequencing of the 16S rRNA gene in a water sample from production well 7,860 taken at the Arkhangelskoe oil field, sulfate-reducing bacteria of the genus *Desulfoplanes* were identified as the dominant group (76% of the nucleotide sequences in the library), and the sulfate-reducing bacterial strain PS50^T^ was isolated (Kadnikov et al., 2023). Preliminary identification of the strain based on the 16S rRNA gene sequencing revealed the highest identity (98.6%) with the gene of the type strain *Desulfoplanes formicivorans* Pf12B^T^, which made it possible to assign strain PS50^T^ to the genus *Desulfoplanes*.

At the time of writing, the genus *Desulfoplanes* was represented only by the type strain Pf12B^T^ of a single species, *Desulfoplanes formicivorans*, isolated from sediment of a meromictic lake located in Japan (Watanabe et al., 2015). The genus *Desulfoplanes* belongs to the family *Desulfomicrobiaceae*, order *Desulfovibrionales*, class *Desulfovibrionia*, of the phylum *Thermodesulfobacteriota*, of the kingdom *Pseudomonadati* (DOI: 10.83108/rn.518744). The family *Desulfomicrobiaceae* comprises three genera, *Desulfomicrobium*, *Thermodesulfomicrobium*, and *Desulfoplanes.* Waite et al. (2020) suggested to assign the genus *Desulfoplanes* to the family *Desulfoplanetaceae*. Bacteria of the genus *Desulfoplanes* are represented by anaerobic, Gram-stain-negative, non-spore-forming vibrions or curved rods that reduce sulfate, thiosulfate, and sulfite to hydrogen sulfide and use a small range of organic compounds or molecular hydrogen + CO_2_ as electron donors and carbon sources. They are incompletely oxidizing organic substrates to acetate.

Given the significant presence of bacteria of the genus *Desulfoplanes* in the oilfield microbial community, the purpose of this work was to determine the phenotypic properties of strain PS50^T^ and its possible involvement in the corrosion of steel equipment, as well as the genes that determine the metabolic activity of *Desulfoplanes* spp. in a subsurface habitat. As a result of the conducted research, the understanding of the phenotypic diversity of the *Desulfoplanes* genus has been expanded. The ability of strain PS50^T^ to cause general corrosion of iron due to sulfide formed in a medium with sulfate and an organic substrate, as well as to cause pitting corrosion in the absence of organic substrates, receiving energy directly from ferrous iron (Fe^2+^) was shown. Detailed genomic and phylogenomic analysis and evaluation of the overall genomic relatedness indices (OGRI) allowed strain PS50^T^ to be assigned to a new species of the genus *Desulfoplanes*, *Desulfoplanes aerotolerans* sp. nov. (VKM B-4014^T^ = KCTC 35101^T^) and identify genes determining the strain’s adaptation to the conditions of the subsurface habitat.

## Materials and methods

### Source of enrichment, media composition, and strain isolation

Strain PS50^T^ was isolated from production water sample 7,860 taken in June 2021 at the Arkhangelskoe oil field (Novosheshminsky district, Tatarstan, Russia) [28]. Coordinates of the sampling point were: 55°13′10.63″N, 51°22′33.94″E. The oil-bearing formations were located at the depth of 830–998 m below sea level, mainly in carbonate rocks, and had a temperature of about 20 °C. The field is operated using water-flooding with a mixture of reservoir water separated from oil and fresh river water to increase the reservoir pressure. Reservoir water samples were of the chlorine-calcium type, characterized by different salinity due to mixing with the injected water (from 36.3 to 244.1 g·L^−1^) and contained high levels of sulfates (0.122–0.708 g·L^−1^). The physicochemical and microbiological characteristics of the oil field were described previously (Nazina et al., 2020; Kadnikov et al., 2023).

Sulfidogenic enrichment 7860E was obtained by inoculating 10% (*w*/*v*) of formation water from production well 7,860 into the anoxic saline mineral medium (SMM) with lactate (4.0 g·L^−1^) and sulfate (2.8 g·L^−1^) as described previously (Kadnikov et al., 2023). This enrichment was further inoculated into media with various substrates and acceptors. The pure culture, designated strain PS50^T^, was obtained after several 10-fold dilutions in the anaerobic liquid medium containing (per liter distilled water): 0.2 g KH_2_PO_4_, 0.25 g NH_4_Cl, 50.0 g NaCl, 3.0 g MgCl_2_·6H_2_O, 0.5 g KCl, 1.5 g CaCl_2_·2H_2_O, 0.5 g cysteine-HCl, and 0.2 g Na_2_S·9H_2_O. The medium was amended with sodium formate (2.0 g·L^−1^), Na_2_S_2_O_3_ (2.0 g·L^−1^), 0.2 g of yeast extract, and 1 mL each of vitamin (Wolin et al., 1963) and microelements solutions (Pfennig and Lippert, 1966). The pH of the medium was adjusted to pH 6.5 with the addition of HCl (10%, *v*/*v*) or NaOH (10%, *w*/*v*) at 22 °C. The media were prepared using the anaerobic Hungate technique (Hungate, 1969) with Ar as a gas phase as described previously (Bidzhieva et al., 2024b). All incubations were at 22 °C unless otherwise noted. The purity of the strain was checked by microscopy of wet biomass and by 16S rRNA gene and genome sequencing as well as was checked in two culture collections, where the strain was deposited (VKM B-4014^T^ = KCTC 35101^T^).

### Phenotypic characterization

Cell morphology, physiological features, DNA isolation, 16S rRNA gene sequencing, and composition of cellular fatty acids and phospholipids were analyzed as described previously [26]. In brief, the temperature range for the growth of the strain PS50^T^ was determined at 5, 10, 15, 20, 25, 30, 37, 42, 48, and 55 °C in a liquid SMM medium containing 50 g NaCl L^−1^, sodium formate (2.0 g·L^−1^) and sodium sulfate (2.0 g·L^−1^), at pH 7.1. The pH range for strain growth was determined in the same medium, but with the addition of buffer solutions; to maintain pH 3.5, 4.0, 4.3, 5.0, 5.6, 6.1, 6.5, 6.8, and 7.5, citrate–phosphate buffer solution (citric acid-Na_2_HPO_4_) was used (Dawson et al., 1986); the pH range of 7.1, 7.5, 8.1, 8.6, and 8.9 was set using a Tris–HCl buffer solution, and the strain was cultured at a temperature of 30 °C. The salinity range for growth was determined in a liquid SMM medium amended with 0, 5, 10, 20, 30, 40, 50, 60, 70, 80, 90, 100, and 120 g NaCl·L^−1^ at a temperature of 30 °C. Additional soluble components (substrates, electron acceptors, etc.) were added into the medium from sterile stock solutions. Substrate utilization tests were performed in SMM medium with sulfate supplemented with 0.1 g·L^−1^ of yeast extract and 2 g·L^−1^ organic substrate each (sodium salts of acetic, pyruvic, succinic, lactic, propionic, benzoic, formic, fumaric, and malic acids, as well as methanol, ethanol, glucose, and yeast extract).

The use of electron acceptors was determined on a SMM medium with Na lactate (2 g·L^−1^) and compared with a control without an electron acceptor. Electron acceptors (Na_2_SO_3_, NaNO_3_, Na_2_S_2_O_3_, Na_2_SO_4_, and S^0^) were introduced into the medium at a concentration of 2 g·L^−1^. Insoluble components (such as S^0^) were added directly into test tubes during the preparation of a liquid nutrient medium. Growth on H_2_/CO_2_ (4,1, *vol./vol.*) was estimated both with and without sodium acetate (0.1 g·L^−1^) as a carbon source. Growth was monitored by optical density (OD_600_) measurements on an Ultrospec 2100 pro spectrophotometer (Amersham Biosciences, Slough, United Kingdom) and by sulfide production, which was determined using the colorimetric method (Trüper and Schlegel, 1964). Nitrite was determined using the Griess reagent.

The ability to ferment organic substrates (glucose, lactate, pyruvate, malate, or/and fumarate) was tested on a medium without electron acceptors. Fermentation products were determined using chromatographic method. Gaseous products of metabolism, H_2_ and CO_2_, were determined by gas chromatography. Volatile fatty acids and alcohols were analyzed on a Shimadzu GC 2010 Plus gas chromatograph (Shimadzu, Kyoto, Japan) in a column (30 m × 0.32 mm) with a Zebron ZB-FFAP phase thickness of 0.25 μm (Phenomenex Ltd., Aschaffenburg, Germany), as described previously (Bidzhieva et al., 2020). The growth of the strain was additionally tested in anaerobic and microaerophilic conditions on SMM medium with sodium lactate and sulfate, containing no cysteine-HCl and Na₂S·9H₂O; the gas phase was argon or air in 5% (*vol./vol.*) O_2_ proportions with argon, respectively. All experiments were carried out in 3 repetitions. All of the incubations were carried out in static conditions at 30 °C for 10 days unless otherwise specified.

Cell morphology, growth, and physiological state of the cultures were assessed also under an Axio 105 Imager. D1 epifluorescence microscope (Carl Zeiss, Oberkochen, Germany). The scanning microscopy images of the cells were obtained using a scanning electron microscope (Quattro S, Thermo Fisher Scientific, Brno-Chernivtsi, Czech Republic) at an accelerating voltage of 15 kV as described previously (Semenova et al., 2024).

For DNA isolation and analyses of cellular fatty acids and polar lipids of the strain PS50^T^, the biomass was obtained in liquid SMM medium with lactate and sulfate after 14 days of incubation at 28 °C. The biomass of the strain was analyzed in the late exponential growth phase; the cells were concentrated by centrifugation for 20 min at 6000g and frozen. The composition of cell wall fatty acids (CFAs) was analyzed using a Maestro gas chromatograph-mass spectrometer (Interlab, Russia) at the All-Russian Collection of Microorganisms (VKM; Pushchino, Moscow Region, Russia) (Bidzhieva et al., 2020). The preparation of the polar lipid extract was carried out according to Minnikin et al. (1984). Polar lipids were separated using two-dimensional thin-layer chromatography on TLC Silica gel 60 (Merck) glass plates in a solvent system of chloroform:methanol:water (65:25:4)—the first direction; chloroform:acetone:methanol:acetic acid:water (50:20:10:10:5)—the second direction (Benning et al., 1995). Lipids were identified as described previously (Nichols, 1963).

The phenotypic features of the strain PS50^T^ were compared with the properties of a type strain of the type species and genus of the family *Desulfomicrobiaceae*, *Desulfomicrobium baculatum* X^T^ (=VKM B-1378^T^ = DSM 4028 ^T^), obtained from the All-Russian Collection of Microorganisms (VKM; Pushchino, Moscow Region, Russia) (Rozanova et al., 1988). The strain X^T^ was grown on the same medium as the PS50^T^ strain, but containing 10.0 g·L^−1^ NaCl. In addition, the characteristics of the strain PS50^T^ were compared with those of the only described species of the genus *Desulfoplanes, Desulfoplanes formicivorans* Pf12B^T^ (=NBRC 110391^T^ = DSM 28890^T^) (Watanabe et al., 2015). Methods for determining the corrosion potential of the strain PS50^T^ are given in the Supplementary material (GOST ISO 9226-2022, 2022).

### DNA isolation and 16S rRNA gene and genome sequencing and annotation

Genomic DNA of strain PS50T was extracted using the Power Soil Pro Kit (Qiagen, Germany). Phylogenetic affiliation was determined by sequencing the 16S rRNA gene fragment amplified with primers 27F and 1492R (Lane, 1991). Sequencing reactions were performed on an automated capillary sequencer ABI Prism 3730 (Applied Biosystems) using the Big Dye Terminator v.3.1 reagent kit. Whole-genome sequencing was carried out using MiSeq (Illumina). Shotgun libraries were constructed with the NEBNext Ultra II DNA Library Preparation Kit (New England BioLabs). The sequencing run generated 9,444,914 paired-end reads (2 × 300 nt), totaling 2,833,474,200 bases. Raw data processing included adapter trimming with Cutadapt v.1.8.3 (Martin, 2011) and quality filtering of read ends using Sickle v.1.33 with a quality threshold of *q* = 20 (Joshi and Fass, 2011). *De novo* genome assembly was performed with SPAdes v.3.15 (Bankevich et al., 2012) using the --isolate parameter. Functional annotation of genetic elements was carried out using the NCBI Prokaryotic Genome Annotation Pipeline (Tatusova et al., 2016).

### Genomic and phylogenetic analyses

Average nucleotide identity (ANI) between genome pairs was calculated using FastANI v1.3 (Jain et al., 2018) with default fragment length (3,000 bp). *In silico* DNA–DNA hybridization (isDDH) values were estimated using formula 2 of the Genome-to-Genome Distance Calculator (GGDC) v3.0 (Meier-Kolthoff et al., 2013, 2022). These metrics collectively constitute the Overall Genome Relatedness Indices (OGRI) (Chun et al., 2018).

For phylogenomic analysis, a set of 120 conserved single-copy bacterial marker proteins (bac120) was identified and aligned using GTDB-Tk v2.1 (Parks et al., 2022). Maximum-likelihood phylogenetic trees were inferred using IQ-TREE2 v2.2 (Minh et al., 2020) under the LG + F + I + G4 substitution model selected by ModelFinder (Kalyaanamoorthy et al., 2017). Neighbor-joining trees were constructed in MEGA11 (Tamura et al., 2021) and maximum-parsimony trees using the MPBoot algorithm (Hoang et al., 2018); bootstrap support was assessed from 1,000 replicates. The same tree-building methods were applied to the 16S rRNA gene alignment, with the GTR + F + G + I model used for maximum-likelihood inference.

A whole-genome-based phylogenetic tree of *Desulfoplanes* members was generated using the CodonTree method within BV-BRC v. 3.58.4 (Wattam et al., 2017), which used PGFams as homology groups. A total of 67 PGFams were found among selected genomes using CodonTree analysis, and the aligned proteins and coding DNA from single-copy genes were used for RAxML analysis (Stamatakis, 2014). iTOL v. 7.2.2 (Letunic and Bork, 2016) was used for tree visualization. The pangenomic analysis was performed using the bioinformatic pipeline IPGA v.1.09 (Liu et al., 2022). The reconstruction of possible metabolic pathways was carried out based on comparison of the genome of strain PS50^T^ using the BlastKOALA v. 3.1 and Mapper v. 5.2 tools of KEGG (Kanehisa et al., 2016), MetaCyc v. 29.6 (Caspi et al., 2020), BRENDA release 2026.1 (Hauenstein et al., 2026), and BV-BRC (Wattam et al., 2017). The neighborhood estimation and visualization of aerotolerance genes were performed using CAGECAT release 1.0 (van den Belt et al., 2023). Gene clusters were drawn using the online service Gene Graphics v. 2.02 (Harrison et al., 2018).

### Nucleotide sequence accession numbers

The GenBank/EMBL/DDBJ accession numbers for the 16S rRNA gene sequence and the genomic assembly of strain PS50^T^ are OQ348127.2 and GCF_053851535.1 (=JBSPZA000000000.1), respectively.

## Results and discussion

### Morphological, physiological, and chemotaxonomic characteristics of the strain PS50^T^

Cells of the *Desulfoplanes* sp. strain PS50^T^ was observed to be Gram-stain-negative motile curved rods or vibrions, 1.8–3.2 μm long and 0.4-0.5 μm in diameter; they did not form spores (Figure 1). Colony formation was not observed in the solid medium with 1.5% agar. The strain PS50^T^ grew in the temperature range of 15–42 °C with an optimum at 25–30 °C, in the NaCl range of 10–100 g·L^−1^ with an optimum at 40–50 g NaCl·L^−1^, at the pH range 5.6–8.6 with an optimum at 7.5 (Supplementary Figure S1). There was no growth during 20 days of incubation at temperatures of 10 and 48 °C, at pH 5 and below and higher than pH 8.9. Thus, it could be considered as a mesophile, neutrophile, and moderate halophile. The main phenotypic features of the strain PS50^T^, which distinguish it from the phylogenetically close species *Desulfoplanes formicivorans*, are listed in Table 1.

**Figure 1:**
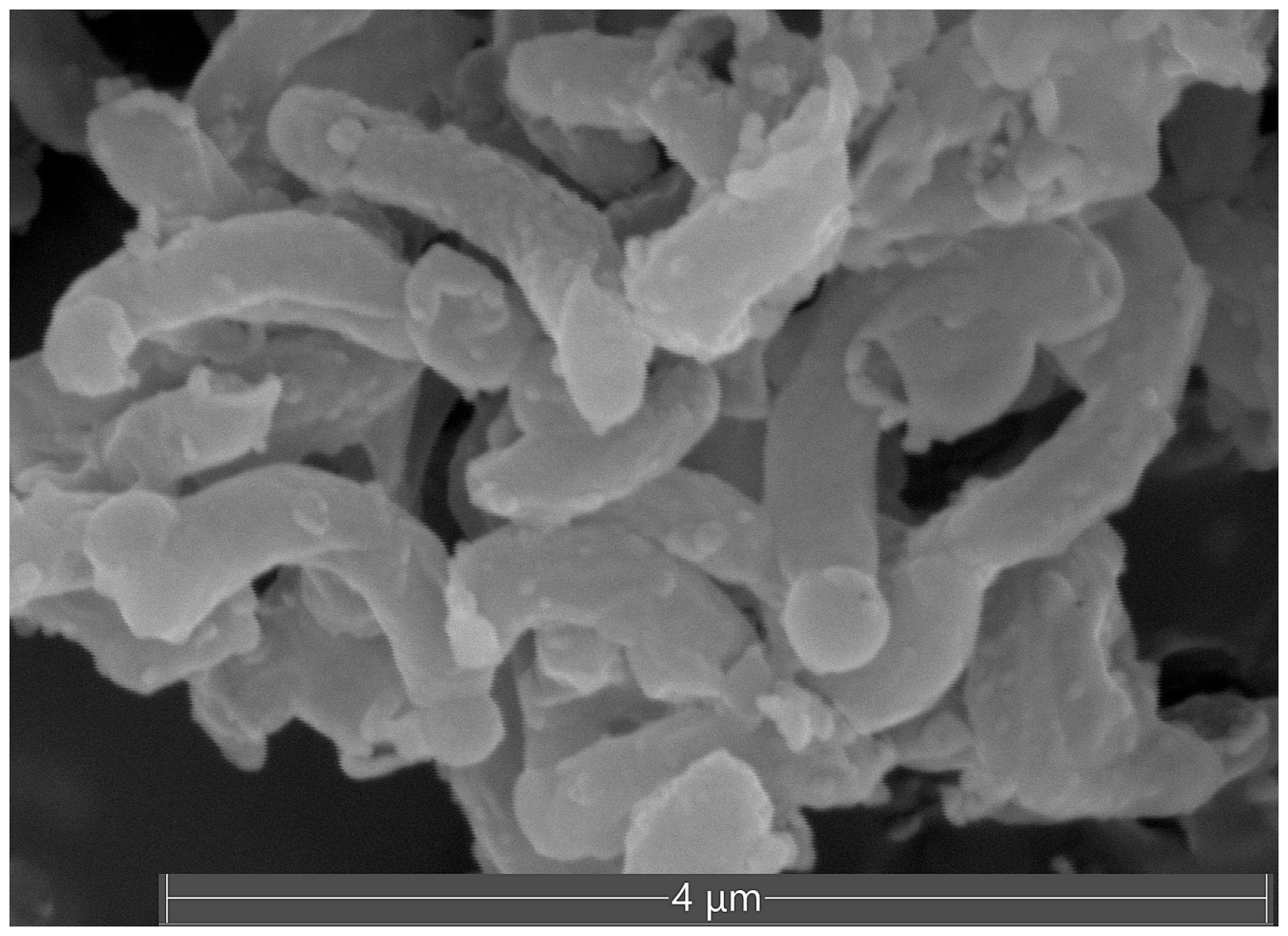
Scanning electron micrograph of total cells of the strain PS50^T^ grown in a medium with lactate and sulfate for 7 days at 30 °C. Bar, 4 μm. A scanning electron microscope (Quattro S, Thermo Fisher Scientific, Brno-Chernivtsi, Czech Republic) at an accelerating voltage of 15 kV.

**Table 1:** Differential characteristics between the strain PS50^T^, *Desulfoplanes formicivorans* Pf12B^T^ and *Desulfomicrobium baculatum* X^T^.

Characteristic	Desulfoplanes aerotolerans*	*Desulfoplanes formicivorans*	*Desulfomicrobium baculatum*
Type strain	PS50^T^	Pf12B^T^	X^T^
Morphology	Rod, vibrio	Vibrio	Rod
Motility	+	+	+
NaCl, min-opt-max, g·L^−1^	10 − (40–50) − 100	5 − (10–40) − 80	0 − 10 − 50
Temperature, min-opt-max, °C	15 − (25–30) − 37	13 − (42–45) − 50	2 − (28–37) − 41
pH, min-opt-max	5.6 − 7.5 − 8.6	6.1 − (7.0–7.5) − 8.6	(7.0–7.5)
Electron donor with sulfate			
H_2_/CO_2_	W	−	W**
H_2_/CO_2_/ацетат	W	+	—
Formate	+	+	+**
Benzoate	−	−	W*
Pyruvate	−	ND	+
Fumarate	+	+	−
Malate	−	ND	+*
Succinate	−	−	W*
Methanol	−	ND	−*
Yeast extract	+	−	−*
Electron acceptor			
Elemental sulfur	+	ND	ND
Fermentation of			
Lactate	W	ND	ND
Glucose	+	−	ND
Malate	W	W	+*
Pyruvate	+	W	W
Genome size (Mb)	3.5	3.0	3.9
Genomic G + C content (mol %)	53.5	55.4	56.8
Major cellular fatty acids (%)	C_17:1_ ω9, i-C_15:0_, ai-C_15:0_	C_16 : 0_, C_18 0_	i-C_17:1_ c7
Isolation source	Petroleum reservoir	Meromictic lake sediment	Сarbonate-manganese ore

*Strain PS50^T^ (data from this study); *D. formicivorans* Pf12B^T^ (Watanabe et al., 2015); *D. baculatum* X^T^ (Rozanova et al., 1988). **Growth is observed in the presence of yeast extract. All taxa are able to utilize lactate, but not acetate, propionate, ethanol, and glucose, as substrates for sulfate reduction and use sulfate, sulfite, and thiosulfate, but not nitrate as electron acceptors. Designations: “+”, positive reaction; (W), weakly positive reaction; “−”, negative reaction; ND, not determined.

In the presence of sulfate as an electron acceptor, strain PS50^T^ was able to grow chemoorganoheterotrophically using formate, lactate, fumarate, and yeast extract, and also grew mixotrophically on a medium with H_2_ and CO_2_ and acetate as carbon sources. Strain PS50^T^ was capable to grow both anaerobically and microaerobically in the presence of 5% atmospheric O_2_. In anaerobic conditions, in a medium with lactate the strain PS50^T^ reduced sulfate, sulfite, thiosulfate, and elemental sulfur with sulfide production. Nitrate was not used as an electron acceptor. The growth on fumarate depended on the presence of sulfate in the medium, which, however, was not reduced to sulfide. In the presence of fumarate and sulfate, up to 8.9% of CO_2_ was revealed in the gas phase, but the accumulation of sulfide in the medium was not detected; without sulfate, the amount of CO_2_ in the gas phase was 0.03%.

The reduction of sulfate to sulfide by strain PS50^T^ was not observed in the presence of benzoate, pyruvate, acetate, propionate, fumarate, malate, succinate, methanol, ethanol, and glucose. Weak fermentative growth was observed with pyruvate, lactate, fumarate, and glucose.

In the cellular fatty acid profile of the strain C_17:1_ ω9 (34.2%), *iso*-C_15:0_ (26.7%), anteiso-C_15:0_ (16.8%), and anteiso-C_17:0_ (7.4%) predominated. Other detected cellular fatty acids are listed in Supplementary Table S1 in comparison with the fatty acid profile of the type strain *Desulfoplanes formicivorans* Pf12B^T^. The predominant components of polar lipids composition of the strain PS50^T^ were phosphatidylethanolamine (PE), phosphatidylglycerol (PG), and diphosphatidylglycerol (DPG). A glycophosholipid (GPL), as well as a number of other aminophosholipids (APL1–3) and phospholipid (PL) were present in trace amounts (Supplementary Figure S2).

### The corrosion potential of the strain PS50^T^

The corrosion rate of steel coupons by the strain PS50^T^ in the presence of sulfate was determined under heterotrophic conditions on an ASW medium with sodium lactate and in mixotrophic conditions when the steel coupon served as a source of electrons, and acetate/CO_2_ were sources of carbon for growth. After 120 days of incubation at 30 °C, very low sulfide formation was observed in mixotrophic conditions, compared with heterotrophic growth (Supplementary Figures S3a,b).

Despite the large differences in H_2_S production during mixotrophic and heterotrophic growth after 20 days of incubation (14 and 517 mg HS^−^·L^−1^, respectively), the corrosion rates were comparable (0.013 and 0.016 mm·year^−1^); on the 120th day of the experiment, the corrosion rate under mixotrophic conditions was 0.025 mm·year^−1^, and exceeded that in a medium with lactate (0.015 mm·year^−1^), despite significant sulfide production in this medium.

Scanning electron microscopy revealed that strain PS50^T^ formed a biofilm associated with a steel coupon (Figure 2a). After removing the biofilms, a pitting corrosion was seen on a steel coupon exposed to SRB (Figure 2b), whereas in a sterile medium, corrosion of the coupon was not observed (Figure 2c).

**Figure 2:**
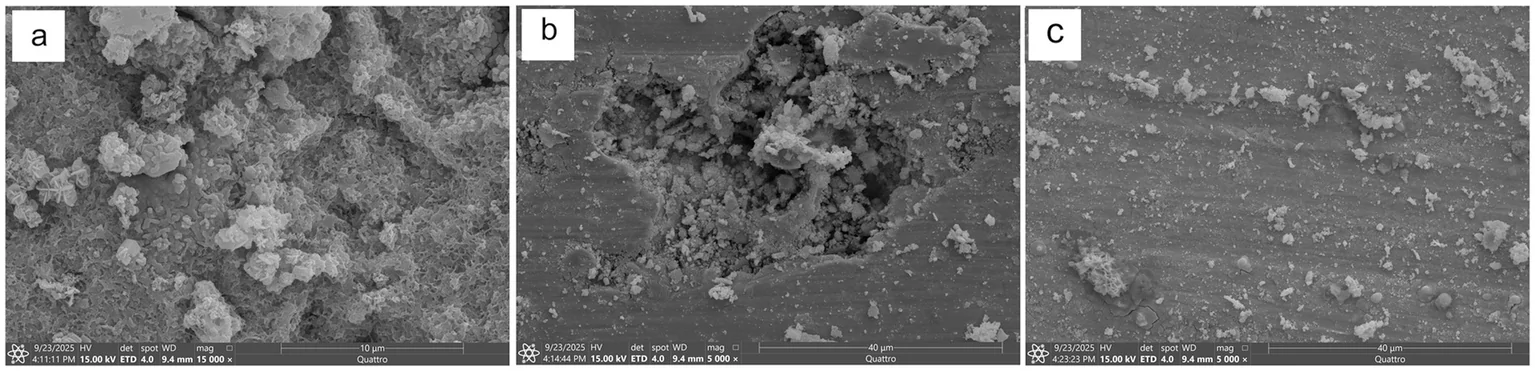
The image of biofilm formation by the strain PS50^T^ on a steel coupon exposed in ASW medium for mixotrophic sulfate-reducing bacteria with acetate (500 mg·L^−1^)/CO_2_ as carbon sources after 100 days of incubation at 30 °C **(a)**. The image of a steel coupon with the pitting corrosion after removing the biofilm **(b)** and sulfide precipitation from it; and of a steel coupon exposed in sterile medium **(c)**. The images were obtained using a scanning electron microscope (Quattro S, Thermo Fisher Scientific, Brno-Chernivtsi, Czech Republic) at an accelerating voltage of 15 kV.

The results of evaluation of the effect of commercial biocides, designated A, B, and C, on the formation of sulfide by the strain PS50^T^ are shown in Supplementary Table S2. The strain PS50^T^ showed low sensitivity to biocides A and B even with a high initial abundance of 10^6^ cells·mL^−1^. Sulfide production was observed at all studied concentrations of biocides A and B, from 40 to 100 mg·L^−1^, whereas a new biocide C, containing polyhexamethylene guanidine hydrochloride, showed high efficiency in suppressing sulfide formation. The above results confirm the need for a preliminary assessment of the effectiveness of biocides on microorganisms in a particular oil reservoir before using these compounds.

### Phylogenetic analysis based on 16S ribosomal RNA gene and genome sequences

The sequence of the 16S rRNA gene of the strain PS50^T^, determined by Sanger sequencing (Acc. no. OQ348127), was identical to the sequence detected in the genome, which confirmed that the genome belonged to the strain PS50^T^. On the phylogenetic tree of 16S rRNA gene sequences, strain PS50^T^ was located in a cluster with the type species of the genus *Desulfoplanes*, *Desulfoplanes formicivorans*, and had 97.5% identity with the respective gene (Figure 3). Uncultured *Desulfoplanes* sp. clone OTU1 from the same habitat belonged to the same cluster with a high level of identity (99.1%) with the 16S rRNA gene of the strain PS50^T^, possibly belonging to the same species as the strain PS50^T^ (Figure 3). This cluster of bacteria of the genus *Desulfoplanes* was located on the same phylogenetic branch as the type species of the family *Desulfomicrobiaceae*, *Desulfomicrobium baculatum* DSM 4028^T^, the level of similarity of nucleotide sequences with the gene of which was only 91.0%, which confirmed the isolated position of the *Desulfoplanes* cluster at the level of a separate genus, proposed by Watanabe et al. (2015). In addition, BLAST analysis revealed a high degree of identity of the 16S rRNA gene (97–99%) of the strain PS50^T^ with the genes identified by molecular analysis of a Guerrero Negro hypersaline microbial mat (Harris et al., 2013; Martínez-Mercado et al., 2025), which indicates the prevalence of *Desulfoplanes* close to the strain PS50^T^ in different high-saline habitats.

**Figure 3:**
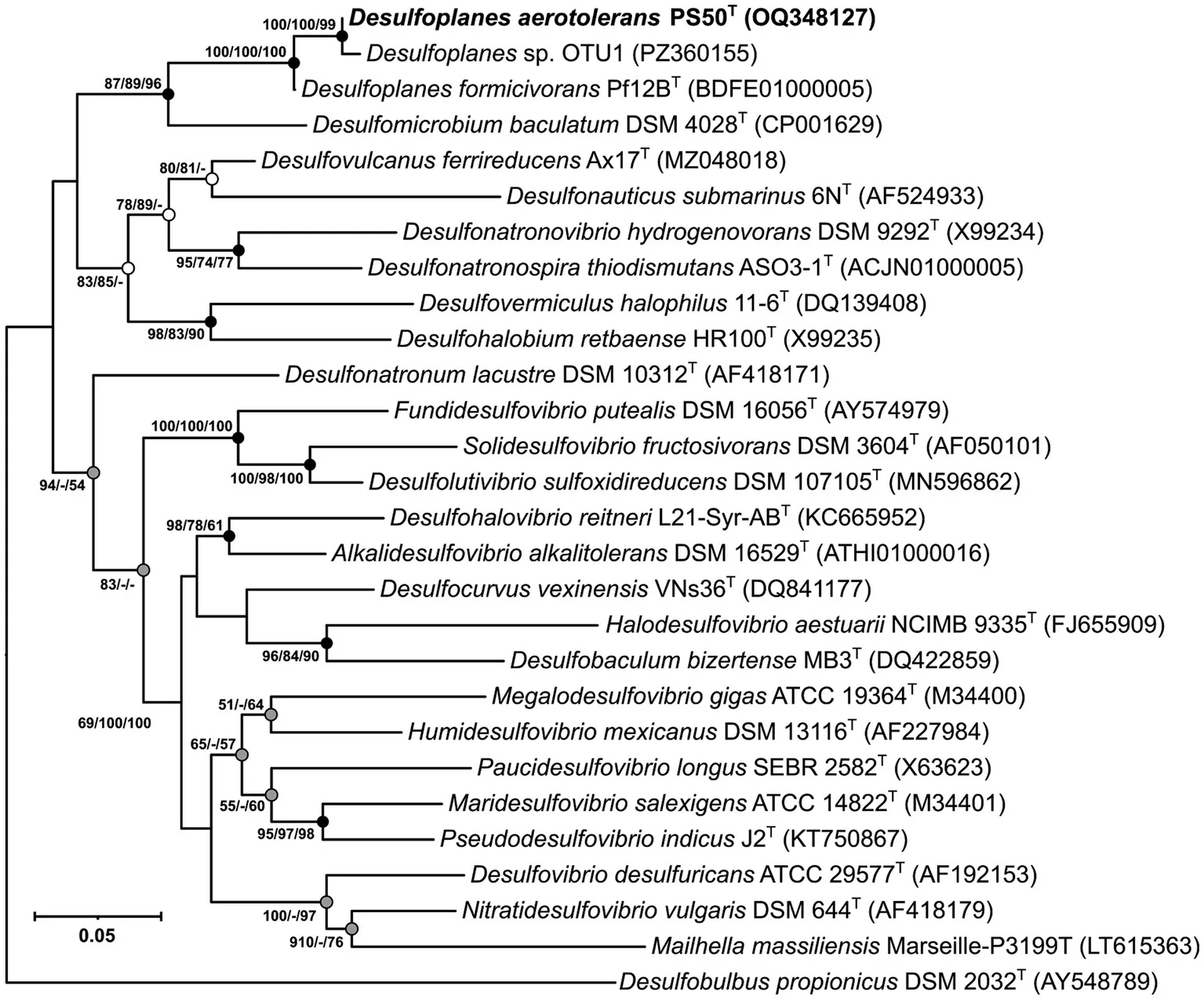
Maximum-likelihood phylogenetic tree based on 16S rRNA gene sequences, showing the position of strain PS50^T^ within the genus *Desulfoplanes* and related taxa. For phylogenetic analysis, 1,506 nucleotide sites were used. White circles indicate the nodes also recovered using the neighbor-joining algorithm; black circles indicate the nodes also recovered using the neighbor-joining and maximum-parsimony algorithms. Bootstrap values (>50%) are listed as percentages at the branching points. Bar, 0.05 substitutions per nucleotide position. The tree was rooted using 16S rRNA gene sequence of *Desulfobulbus propionicus* DSM 2032^T^ as the outgroup. GenBank accession numbers for 16S rRNA gene sequences are indicated in parentheses.

To clarify the taxonomic position and possible ecological function of the strain PS50^T^ in petroleum reservoirs with high-salinity formation water, its genome was sequenced and analyzed (BioProject accession number PRJNA1308096). Maximum-likelihood, neighbor-joining, and maximum-parsimony phylogenomic trees based on a set of 120 conserved single-copy bacterial marker proteins [120bac, 53] were consistent with the 16S rRNA gene sequences tree (Figure 4). On the phylogenomic tree the strain PS50^T^ formed a distinct cluster with 100% bootstrap value with *D. formicivorans* Pf12B^T^ and metagenome-assembled genome MAG IW1_01 from the injection water IW1 of the Sabanchinskoe oil field (Kadnikov et al., 2023).

**Figure 4:**
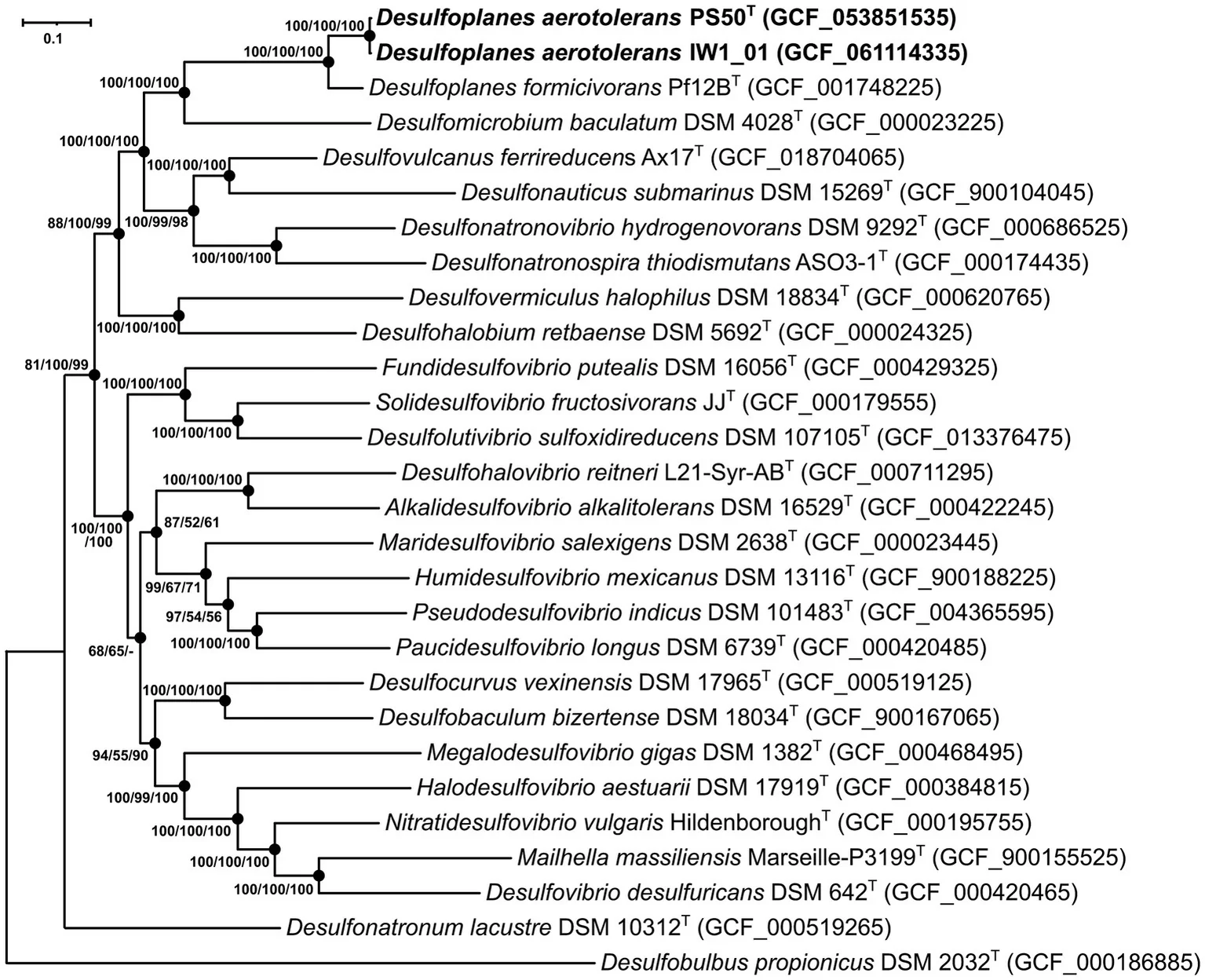
Maximum-likelihood phylogenetic tree based on concatenated bac120 single-copy marker proteins, showing the position of strain *Desulfoplanes aerotolerans* PS50^T^ within the genus *Desulfoplanes* and related taxa. For phylogenetic analysis, 34,747 amino acid positions were used. Black circles indicate the nodes also recovered using the neighbor-joining and maximum-parsimony algorithms. Bootstrap values (>50%) are listed as percentages at the branching points. Bar, 0.1 amino acid substitutions per site. The tree was rooted using *Desulfobulbus propionicus* DSM 2032^T^ as the outgroup. Accession numbers for genomic assemblies are indicated in parentheses.

The ANI and dDDH values between the PS50^T^ genome and the genome of closely related type strain *D. formicivorans* Pf12B^T^ were 80.1 and 22.3%, respectively. These values are below the thresholds for species delineation of 95–96% for ANI and 70% for dDDH (Meier-Kolthoff et al., 2013; Chun et al., 2018); this confirms that they belong to different species of the genus *Desulfoplanes*.

The genomes of SRB of family *Desulfomicrobiaceae*, including type strains of described species of the genera *Desulfomicrobium*, *Thermodesulfomicrobium*, as well as the PS50^T^, Pf12B^T^ and IW1-1 genomes of *Desulfoplanes*, were used in a pangenome analysis conducted using the IPGA service (Supplementary Figure S4). All strains in this family could be divided into three groups based on the value of ANI (Supplementary Figure S5). Furthermore, the hierarchical structure of the ANI result and pangenome profile were identical to the polygenetic trees created based on 16S rRNA genes and genomes. Among 15,029 orthologous gene clusters, only 241 (1.6%) were core gene clusters. It is worth noting that key sulfate-reduction genes—*sat*, *apr*AB, and *dsr*AB are annotated as core genes. 354–355).

### Metagenomic analysis of PS50^T^-related strains in environment and genomic databases

To date, the GenBank database contains 10 genomes identified as belonging to bacteria of the genus *Desulfoplanes*. However, only two of these belong to strains isolated in pure culture—the type strain *D. formicivorans* Pf12B^T^ and the new strain PS50^T^—while the others are metagenome-assembled genomes (MAGs) from various habitats. MAG IW1_01, identified through the analysis of formation water from an oil reservoir in Tatarstan, has not yet been deposited in GenBank and currently holds only a BV-BRC ID (1634037.5).

To confirm the phylogenetic position of the strain PS50^T^ among *Desulfoplanes* with high robustness, a phylogenetic tree was constructed based on 67 single-copy proteins using the Bacterial Genome Tree Service of the BV-BRC portal (Supplementary Figure S6). Eleven genomes of the genus *Desulfoplanes*, including the genomes of all *Desulfoplanes* and genomes of some type strains of other SRB were used for analysis. In the phylogenomic tree, strain PS50^T^ was included in a common cluster with the MAG-genomes IW1_01 from the oilfield and E22bin.0671 (Acc. num. JBUDSQ010000000) which was assembled from pool of DNA from hypersaline microbial mat cores incubated with hydrogen, carbon dioxide, and trimethylamine (Guerrero Negro, Baja California Sur, Mexico) (Martínez-Mercado et al., 2025). Analysis of ANI similarity between the PS50^T^ genome and those of other *Desulfoplanes* genomes (Supplementary Figure S7) showed its highest similarity with the MAG IW1_01 (98.6%), suggesting they may belong to the same species. MAG *Desulfoplanes* sp. E22bin.0671 was more closely related to the PS50^T^ genome (ANI value 88%) than to all other *Desulfoplanes* metagenomes (72.5–79.7%).

These results indicate the presence of bacteria of the genus *Desulfoplanes*, similar to the isolated strain, in petroleum reservoirs with highly saline waters and in hypersaline microbial mat cores.

### Genomic analysis

In this study, a genomic analysis was conducted to describe the general genomic features of bacteria of the genus *Desulfoplanes* and specific features that distinguish the strain PS50^T^ from the type species *Desulfoplanes formicivorans* and others uncultured *Desulfoplanes* spp. The genome of the strain PS50^T^ consists of 3,501,441 bp and comprises 93 scaffolds, with an N_50_ value of 78,691 bp, a G + C content of 53.5%, and coverage of 421×. The genome exhibited 98.81% completeness and 0.079% contamination; these values were within the accepted thresholds for high-quality genomes (≥90% completeness and ≤5% contamination), as proposed by Parks et al. (2020). Using the PGAAP in the genome sequence of PS50^T^, were predicted 3,181 genes, of which 3,078 were protein-coding sequences, 32 were pseudo genes, and 71 RNAs genes.

Analysis of enzyme composition of the metabolic pathways based on the results of functional protein prediction using the BlastKOALA and BV-BRC services provides evidence that the genome of strain PS50^T^ contains all the key genes responsible for complete pathways of central carbohydrate metabolism: glycolysis (the Embden-Meyerhof pathway)/gluconeogenesis; pentose phosphate pathway, pyruvate oxidation and 5-phospho-alpha-D-ribose 1-diphosphate (PRPP) biosynthesis. The citrate cycle (TCA) of strain PS50^T^ is not closed; only the enzymes of first carbon oxidation are fully represented. A complete set of genes only for F-type ATPase (ЕC: 7.1.2.2) has been identified among enzyme complexes of ATP synthesis. Complete sets of the genes were revealed, which were responsible for dissimilatory sulfate reduction, biosynthesis of basic amino acids, biosynthesis and beta-oxidation of fatty acids, phosphatidylethanolamine (PE) biosynthesis, some nucleotide sugar biosynthesis, C5- and C10-isoprenoid biosynthesis, as well as the biosynthesis of vitamins and cofactors: pantothenate; heme; and menaquinone (futalosine pathway). At the same time, the genes encoding enzymes of assimilatory and dissimilatory nitrate reduction, as well as complete xenobiotic degradation pathways have not been identified.

The genome of strain PS50^T^ was compared with that of its closest validly described relative, *D. formicivorans* Pf12B^T^. The PS50^T^ genome contained 3,078 predicted proteins, of which 1,559 (50.6%) were assigned KEGG Orthology (KO) terms (Table 2). In comparison, *D. formicivorans* Pf12B^T^ contains 2,657 predicted proteins, including 1,414 proteins with KO assignments (53.2%). Thus, PS50^T^ has a larger predicted gene complement and a slightly larger KO repertoire than *D. formicivorans*. At the KO level, 1,059 terms were shared between the two genomes, whereas 165 KOs were detected only in PS50^T^ and 96 only in *D. formicivorans*. These numbers indicate that the two organisms share the major functional framework expected for members of *Desulfoplanes*, but that PS50^T^ also carries a distinct accessory functional repertoire.

**Table 2:** General KEGG-based genomic features of strain PS50^T^ and *D. formicivorans* Pf12B^T^.

KEGG-based genomic feature	Genome of	
	Strain PS50^T^	Strain Pf12B^T^
Predicted proteins in KO	3,078	2,657
Proteins with KO assignment	1,559 (50.6%)	1,414 (53.2%)
Unique KO terms detected	1,224	1,155
Shared KO terms	1,059	1,059
Strain-specific KO terms	165	96
Proteins without primary KO assignment	1,519 (49.4%)	1,243 (46.8%)

The central anaerobic energy metabolism of PS50^T^ broadly resembles that of *D. formicivorans*, but several differences suggest additional metabolic flexibility. PS50^T^ contains primary KEGG annotations for pyruvate dehydrogenase components *pdh*A, *pdh*B, and *pdh*C/*ace*F, whereas these components were not detected as primary KO assignments in *D. formicivorans* Pf12B^T^ (Supplementary Table S3). This gene set may support pyruvate oxidation under redox-variable conditions and is consistent with the aerotolerant phenotype, although physiological evidence remains essential for this interpretation.

Both organisms encode pyruvate:ferredoxin/flavodoxin oxidoreductase (*por*/*nif*J, K03737), indicating that anaerobic pyruvate metabolism is conserved. PS50^T^ also carries a broader set of carbon monoxide dehydrogenase-associated annotations, including CooF, while *D. formicivorans* Pf12B^T^ genome has more formate dehydrogenase subunit annotations and formate-tetrahydrofolate ligase. This contrast is consistent with the known formate-associated physiology of *D. formicivorans* and suggests that PS50^T^ may rely on a somewhat different balance of electron donors and redox carriers.

PS50^T^ also contains a broader set of primary NADH-quinone oxidoreductase annotations, including *nuo*C and *nuo*I, compared with the KO profile of Pf12B^T^ genome. This pattern indicates differences in the annotated respiratory electron-transport repertoire and may reflect variation in respiratory-chain organization between the two strains.

#### Conserved sulfate-reducing metabolism

Both genomes, PS50^T^ and Pf12B^T^, encode the canonical machinery for dissimilatory sulfate reduction (Supplementary Figure S8). The sulfur metabolism genes in the PS50^T^ genome are shown in Supplementary Figure S9. Shared functions include sulfate adenylyltransferase Sat (K00958), adenylylsulfate reductase AprAB (K00394/K00395), dissimilatory sulfite reductase DsrAB (K11180/K11181), DsrC, and the membrane-associated DsrMKJOP complex (Supplementary Table S3). This conserved gene set supports the placement of PS50^T^ among sulfate-reducing *Desulfoplanes* and indicates that sulfate respiration is a core trait shared with *D. formicivorans*.

According to the KEGG map of sulfur metabolism, the reduction of sulfate to sulfide by strain PS50^T^ is carried out only via a dissimilatory process (Supplementary Figure S8). The genes determining sulfate reduction to sulfite are represented by a cluster including the *sat* gene (ACTVJH_06650) encoding sulfate adenylyltransferase (EC: 2.7.7.4), *apr*AB genes (ACTVJH_06655-06660), coding two adenylylsulfate reductase subunits (EC: 1.8.99.2), as well as *qmo*A gene (ACTVJH_06655), coding the QmoA protein, which is presumably part of the anaerobic electron transfer complex QmoABC associated with adenylylsulfate reductase, incomplete due to a sequence break (gap). It has been proposed that Qmo-complex transfers electrons from the quinone pool to AprAB, in a process that may result in energy conservation (Pires et al., 2003; Venceslau et al., 2010). Genomic analysis confirmed that a gene locus containing *sat*, *apr*AB, and the *qmo*ABC genes is present in the majority of SRB analyzed (Pereira et al., 2011). Further reduction of sulfite to sulfide via the intermediate metabolite [DsrC-protein]-trisulfide is carried out by the enzyme dissimilatory sulfite reductase (EC: 1.8.1.22), the subunits of which are determined by the *dsr*ABD gene cluster (ACTVJH_02060–02050) and [DsrC]– trisulfide reductase (EC: 1.8.5.10). The latter is encoded by the complete cluster of genes of the *dsr*MKJOP (ACTVJH_02105–02085) sulfite reduction-associated complex and the *dsr*C gene (ACTVJH_11480) encoding sulfite reductase associated protein, but localized in another part of the genome.

To analyze the evolutionary characteristics of key genes involved in dissimilatory sulfate reduction in strain PS50^T^, a phylogenetic tree was constructed based on the translated amino acid sequences of *dsr*AB genes from SRB of the family *Desulfomicrobiaceae*; this analysis included type strains of the genera *Desulfomicrobium*, *Thermodesulfomicrobium*, and *Desulfoplanes*, as well as several *Desulfoplanes* MAGs (metagenome-assembled genomes) from various environments (Supplementary Figure S10). The taxonomic distribution of the analyzed sequences was congruent with trees based on 16S rRNA genes and whole-genome sequences, 460 that *dsr*AB evolution within the *Desulfomicrobiaceae* family was driven by vertical inheritance—although literature reports indicate multiple horizontal gene transfer events in distinct SRB lineages (Tao et al., 2024).

Of the genes encoding enzymes of assimilatory sulfate reduction in the PS50^T^ genome were present the genes coding enzymes of the first stage of this process—the reduction of sulfate to sulfite, namely, the *cys*NC gene (ACTVJH_01565) encoding a bifunctional enzyme adenylyl-sulfate kinase/sulfate adenylyltransferase (EC: 2.7.1.25/2.7.7.4). An orthologous set of genes for sulfur metabolism is present in the genome of *D. formicivorans* Pf12B^T^.

#### Carbon metabolism

In the presence of sulfate, the strain PS50^T^ uses a limited set of substrates for growth, including lactate, formate, and H_2_/CO_2_/acetate.

*Lactate/Pyruvate*: The oxidation of lactate to pyruvate and then to acetate is a key metabolic process in SRB. The electrons produced as a result of this process are used to carry out the dissimilatory reduction of sulfate, which generates cellular energy in the form of ATP. The stages and genetic determinants of this process have been described in the greatest detail for members of the genus *Desulfovibrio*. In the genome of the model type strain (*Nitrati*) *Desulfovibrio vulgaris* Hildenborough, the genes determining this process are grouped into the luo-operon (short for Lactate Utilization Operon) (DVU3025–3033), which is a nonacistronic transcriptional unit. Homologous gene structures have also been identified in the genomes of other *Desulfovibrio* species and strains (Vita et al., 2015). The luo-operon comprises the genes encoding enzymes involved in different stages of the oxidation process: transmembrane transport of lactate into the cell, followed by its oxidation via pyruvate and acetyl-CoA to acetate and CO₂.

According to the KEGG map “Pyruvate Metabolism” (Supplementary Figure S11), the genome of the strain PS50^T^ contains all the enzymes involved in the utilization of lactate and pyruvate to form acetate and CO₂. However, the genes responsible for this process are not organized into a single luo-operon but are distributed across different regions of the genome, and some of them are duplicated (Figure 5). The predicted genes *por*-I (ACTVJH_14300) encoding pyruvate:ferredoxin oxidoreductase (PFOR) (EC: 1.2.7.1), *lld*P (ACTVJH_14305) encoding D, L-lactate permease, and *dld*II-AB (ACTVJH_14310–14,315) encoding quinone-dependent D-lactate dehydrogenase (EC: 1.1.5.12) are grouped into a single cluster and are homologous to the corresponding genes of the luo-operon of *N. vulgaris* Hildenborough (DVU3025–3028), with a translated amino acid sequence identity of 74%, suggesting similar functionality. However, the *pta* and *ack*A genes in the luo-operon of *N. vulgaris* Hildenborough, which encode phosphate acetyltransferase and acetate kinase, respectively, and convert acetyl-CoA into acetate with ATP formation, are not annotated in the genome of strain PS50^T^. They may be replaced by the *acs* genes (ACTVJH_14320; ACTVJH_07150), which encode putative acetyl-CoA synthetase (ADP-forming) (EC: 6.2.1.1); however, this hypothesis requires further investigation, as the structure and function of this enzyme have so far been described only for archaea (Jones et al., 2017). Homologous to the remaining genes of the *N. vulgaris* Hildenborough luo-operon (DVU2032–2033), the *lld*GH genes (ACTVJH_06670–06675), which are presumed to encode L-lactate dehydrogenase (quinone) (EC: 1.1.2.3), are arranged in a separate tandem cluster.

**Figure 5:**
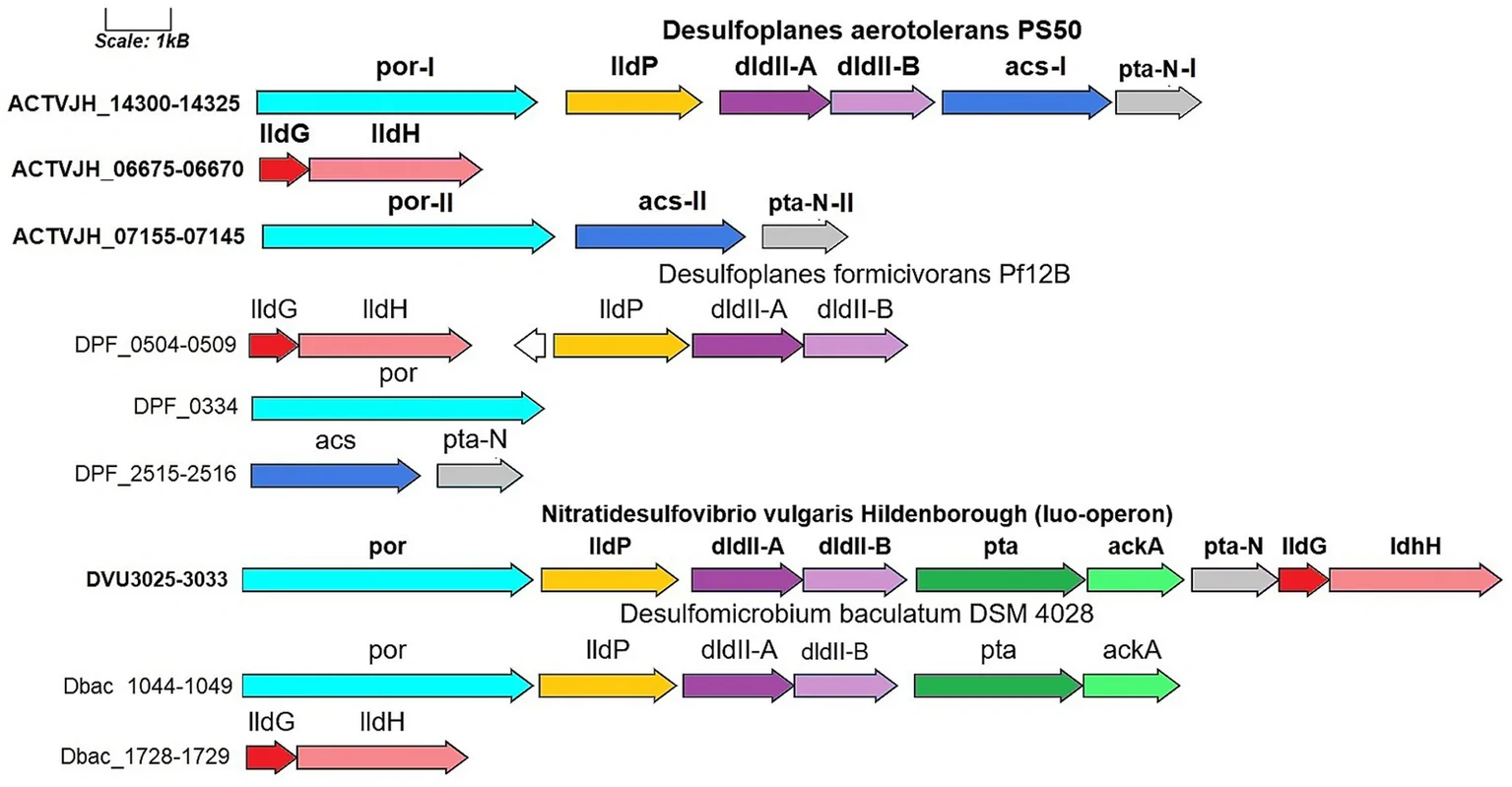
Comparison of the structure of genome regions containing presumably annotated lactate-pyruvate utilization genes in the type strains of *Desulfoplanes aerotolerans* PS50^T^, *D. formicivorans* Pf12B^T^, *Nitratidesulfovibrio vulgaris* str. Hildenborough^T^ and *Desulfomicrobium baculatum* DSM 4028^T^. Abbreviations: *por*, pyruvate-flavodoxin oxidoreductase; *lld*P, L-lactate permease; *dld*II-A, D-lactate dehydrogenase (cytochrome); *dld*II-B, D-lactate dehydrogenase, iron–sulfur subunit; *pta*, phosphate acetyltransferase; *ack*A, acetate kinase; *acs*, putative acetyl-CoA synthetase (ADP-forming); *pta*-N, N-terminal domain of phosphotransacetylase (function is unknown); *lld*H, L-lactate dehydrogenase (quinone) large subunit; *lld*G, L-lactate dehydrogenase, iron–sulfur cluster-binding protein.

The genome of the type strain *D. formicivorans* Pf12B^T^ also contains all genes homologous to those of the *N. vulgaris* Hildenborough luo-operon; however, their localization in the genome differs significantly from that in the strain PS50*^T^* (Figure 5). Unfortunately, it is not possible to analyze the lactate and pyruvate utilization genes in the MAGs of *Desulfoplanes* strains available in the databases due to incomplete sequencing.

In addition to the differences between the genomes of strain PS50^T^ and *D. formicivorans* Pf12B^T^ mentioned above, there are several others related to the KEGG map “Pyruvate Metabolism” (Supplementary Figure S11). Exclusively in the PS50^T^ genome, the *pdh*AB genes (ACTVJH_06045–06055) are tentatively annotated, encoding the enzyme pyruvate dehydrogenase (acetyl-transferring) (EC: 1.2.4.1); *pdh*C, which encodes dihydrolipoyllysine-residue acetyltransferase (EC: 2.3.1.12); and *pdh*D, which encodes dihydrolipoyl dehydrogenase (EC 1.8.1.4). It is generally accepted that these enzymes are the E1–E3 components of a single pyruvate dehydrogenase (PDH) complex, which converts pyruvate to acetyl-CoA and is specific to aerobic prokaryotes and eukaryotes (Škerlová et al., 2021). How does this complex function in the cells of the strain PS50^T^ is still unknown without special studies. However, since the strain under study does not utilize pyruvate as an electron donor, it can be assumed that the enzymes of aerobic pyruvate oxidation are used not for sulfate respiration, but as an additional mechanism of oxygen detoxification, which promotes the active growth of this bacterium under microaerophilic conditions. At the same time, unlike the type strain of the species *D. formicivorans* Pf12B^T^, the genome of the strain PS50^T^ under study does not contain the *pfl*D gene encoding the enzyme pyruvate formate-lyase (EC: 2.3.1.54), which ferments pyruvate to acetyl-CoA and formate. However, its genome presumably contains the genes auxiliary to this process, pyruvate formate lyase activating enzyme (EC: 1.97.1.4), which may indicate the loss of the key gene *pfl*D during evolution.

*Fumarate, succinate, malate*: Although the strain PS50^T^ cannot grow with succinate and malate as electron donors and grows only weakly on fumarate, the genes to metabolize these compounds to pyruvate are present in its genome. In the genome of the studied strain, putatively coding genes *frd*CAB (ACTVJH_01525–01435) for fumarate reductase (EC: 1.3.5.1), transferring electrons from the quinol pool to fumarate and participating in anaerobic respiration with fumarate as the terminal electron acceptor (Guan et al., 2018); the genes *fum*AB (ACTVJH_01540–015450) for fumarate hydratase (EC: 4.2.1.2), which facilitates the reversible conversion of fumarate to L-malate; *mae*B (ACTVJH_01755), and the gene for NADP^+^-dependent malic enzyme (EC: 1.1.1.40), which catalyzes the oxidative decarboxylation of L-malate into pyruvate. Homologous genes were annotated in both the genome of the type strain *D. formicivorans* Pf12B^T^ and the MAGs of *Desulfoplanes* strains.

*Formate*: The ability of strain PS50^T^ to use formate as an electron donor during anaerobic sulfate reduction is confirmed by the annotation of genes in its genome that putatively encode three formate dehydrogenases (FDHs). The genes *fdh*AB_1 (ACTVJH_05480–05485) and *fdh*AB_2 (ACTVJH_01825–01830) encode the enzymes conferring selenocysteine-incorporated metal-dependent formate dehydrogenases in reactions involving NAD+ (EC: 1.17.1.9) and NADP+ (EC: 1.17.1.10), respectively. These enzymes catalyze the oxidation of formate into CO_2_, generating both H_2_ and NADH (NADPH), or, under certain physiological conditions, can operate in reverse to reduce CO_2_ to formate (Oliveira et al., 2026). In the genome, the *fdh*AB_1 tandem is flanked by the accessory genes *fdh*D (ACTVJH_05475) of formate dehydrogenase accessory sulfurtransferase and *moe*A (ACTVJH_05470) of molybdopterin molybdotransferase (EC: 2.10.1.1), a specialized protein essential for the maturation and activation of molybdenum- and tungsten-containing FDHs. Homologous genes are also annotated in the *D. formicivorans* Pf12B^T^ genome and some MAGs of *Desulfoplanes*, which apparently indicates their necessity for the metabolism of SRB in this genus. However, only the genome of strain PS50^T^ contains annotated genes *fdh*AB_3 (ACTVJH_06710–06715), which possibly encode the coenzyme F420-dependent FDH (EC: 1.17.98.3). However, this enzyme has only been characterized in methanogenic archaea, so its structural and functional properties in this strain require further study.

*СО*: Carbon monoxide (CO) is a gas that is toxic to various organisms, including humans and even microbes; however, it has a low redox potential, which can fuel certain microorganisms, namely, CO oxidizers (Fukuyama et al., 2020; Katayama et al., 2026). Many SRBs are able to use carbon monoxide as a carbon source and electron donor for biological sulfate reduction, although they vary significantly in their tolerance to CO toxicity. The most resistant strains can use CO as the sole electron donor (at a CO concentration of 100% in the gas phase), while the growth of others is significantly inhibited at CO concentrations below 1-2% (Parshina et al., 2010). The key enzyme for anaerobic CO utilization is a nickel-iron [Ni-Fe] carbon monoxide dehydrogenase (CODH) (EC: 1.2.7.4), catalyzing the reversible conversion of CO and CO_2_, which in SRBs is predominantly directed toward CO oxidation. This enzyme is also key to the reductive acetyl-CoA pathway (the Wood-Ljungdahl pathway), as in *Desulfofundulus salinus* 435^T^, which was previously isolated and studied (Nazina et al., 2024). It is capable of growing on carbon monoxide, although it does not grow on CO as a sole substrate, but requires hydrogen as an electron donor. Although the genome of strain PS50^T^ does not contain genes for the complete Wood-Ljungdahl pathway, it does contain duplicated genes for the *coo*S (ACTVJH_07780; ACTVJH_14200), the main catalytic subunit of carbon monoxide dehydrogenase CooS (EC: 1.2.7.4), as well as auxiliary genes *coo*F (ACTVJH_14205) for the CO dehydrogenase iron–sulfur protein CooF (EC: 1.2.7.4) and *coo*C (ACTVJH_14195) for accessory protein CooC (nickel insertion), suggesting that the strain under study can grow on CO. In the type strain *D. formicivorans* Pf12B^T^, only *coo*SC genes are annotated in the genome, so their functionality is questionable.

#### Aerotolerance, redox stress, and osmoprotection

Due to the sensitivity of the sulfate-reducing enzymes to oxygen, SRB are traditionally classified as strict anaerobes. However, they are often found and even dominant in natural habitats where oxygenic conditions may temporarily or periodically arise, such as in marine intertidal sediments, microbial mats, marshes, and also in oil reservoirs, where dissolved oxygen enters with injected water (Nazina et al., 2017; Dyksma and Pester, 2024). This is facilitated by the presence of a complex system of defense mechanisms, allowing them to survive and even reproduce in periodically aerobic environments. The set of genes determining oxygen tolerance mechanisms has been studied in most detail for bacteria of the genus *Desulfovibrio* (Dolla et al., 2006). The genes determining key factors from this set are also annotated in the genome of strain PS50^T^. The potential ability of strain PS50^T^ to directly reduce oxygen is suggested by the presence in its genome of two types of reductase genes that reduce oxygen to water. The *roo/nor*V gene (ACTVJH_06815) specifies a cytoplasmic bifunctional rubredoxin:oxygen oxidoreductase/nitric oxide reductase, which functions with the participation of rubredoxin. The *cyd*AB gene tandem (ACTVJH_06755-06760) presumably encodes a membrane-bound oxygen reductase such as cytochrome bd ubiquinol oxidase (EC: 7.1.1.7). According to the literature, this enzyme is known to play an important role in oxygen consumption as a defense mechanism (Pereira et al., 2011; Ramel et al., 2013).

The genome of strain PS50^T^ also contains annotated genes encoding enzymes that detoxify the toxic reactive oxygen species (superoxide and hydrogen peroxide) formed upon contact with oxygen inside the cell. Superoxide can be disproportionated by superoxide dismutase, Fe-Mn family (EC: 1.15.1.1) to molecular oxygen and hydrogen peroxide and is encoded by the *sod*B gene (ACTVJH_06780). The *kat*A gene (ACTVJH_00065) presumably encodes catalase (EC: 1.11.1.6), which catalyzes the disproportionation of H_2_O_2_ to H_2_O and O_2_, thus protecting the cells against the oxidative effects of H_2_O_2_. The activity of this enzyme was previously demonstrated for SRBs of the genera *Desulfovibrio* and *Desulfomicrobium* (Kushkevych et al., 2014). A synergistic effect between superoxide dismutase and catalase has also been previously demonstrated, significantly increasing oxidative resistance in the facultatively anaerobic *Lactobacillus rhamnosus* (An et al., 2011), which may also be characteristic of other anaerobes. Furthermore, the *rbr* gene (ACTVJH_06830) was annotated in the genome of the studied strain, presumably encoding the protein rubrerythrin, which also promotes hydrogen peroxide elimination. Expression of genes in this cluster is presumably regulated by the *per*R gene (ACTVJH_06835), encoding the PerR protein (Peroxide Resistance Regulator), which acts as a specialized metal-dependent transcriptional repressor that controls oxidative stress and iron homeostasis (Zhou et al., 2010).

Homologous genes encoding enzymes that protect against the toxic effects of oxygen and its reactive species are also annotated in the genome of the type strain of *D. formicivorans* Pf12B^T^, as well as in those MAGs of *Desulfoplanes* in which the corresponding regions have been sequenced. Most of these genes were included in a single gene cluster; many of these, however, have not yet been annotated (Figure 6). According to data from the CAGECAT portal, this cluster is specific only to SRB of the genus *Desulfoplanes*.

**Figure 6:**
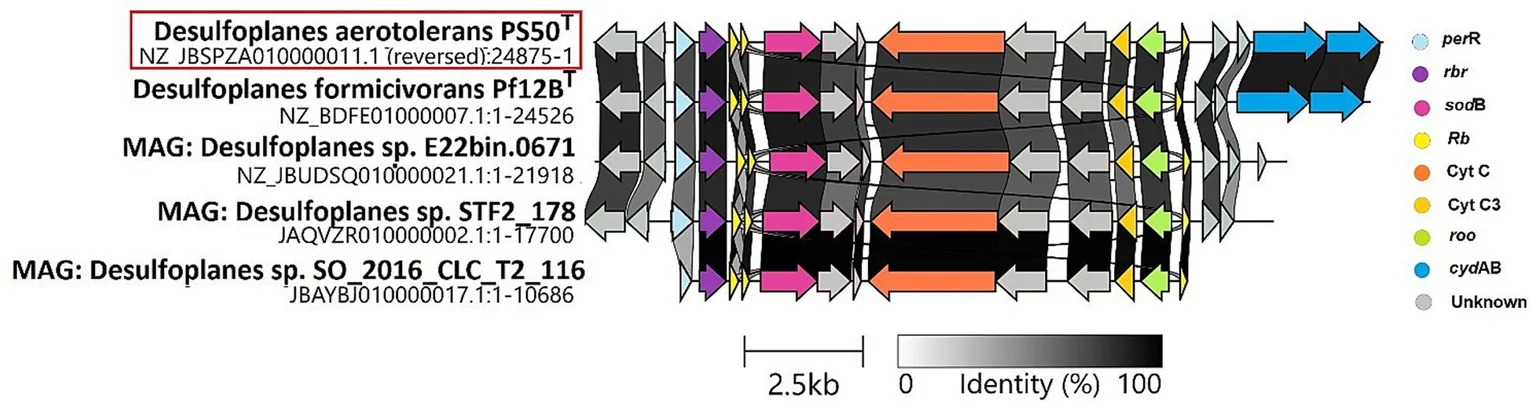
Gene synteny of oxygen resistance in the PS50^T^ genome and genomes of *Desulfoplanes* strains and MAGs. Abbreviations: *per*R, peroxide stress regulator PerR, FUR family; *rbr*, rubrerythrin; *sod*B, superoxide dismutase, Fe-Mn family; *Rb*, rubredoxin; *Cyt C*, cytochrome c family protein; *Cyt C3*, cytochrome c3 family protein; *roo*, rubredoxin:oxygen oxidoreductase/nitric oxide reductase; *cyd*AB, cytochrome d ubiquinol oxidase.

In addition to oxygen detoxification mechanisms, many SRBs may have mechanisms for repairing damaged proteins. Strain PS50^T^ is believed to possess several enzymatic systems. Its genome contains annotated genes and gene clusters d*na*J*-daf*A*-clp*B (ACTVJH_02395-02385), *dna*K (ACTVJH_08270), and *gro*ES*-gro*EL (ACTVJH_02315-02320), encoding the ClpB-DnaK and GroEL/GroES chaperone systems. These systems ensure proper folding and prevent the aggregation of denatured proteins and are conserved across SRBs, including members of *Desulfoplanes* (Rosenzweig et al., 2013; Ishii, 2017). Another mechanism for repairing protein damage resulting from oxidative stress is the action of the enzyme methionine sulfoxide reductase, which converts oxidized methionine (MetSO) back to its functional form, methionine. The genes *msr*A and *msr*B encode two forms of the enzyme, reducing S-MetSO and R-MetSO, respectively, which differ in structure, active site composition, and protein folding (Ezraty et al., 2017). The *mtr*B and *mtr*A/B genes, encoding peptide-methionine sulfoxide reductase MsrB (EC: 1.8.4.12) and bifunctional peptide methionine sulfoxide reductase MsrA/MsrB (EC: 1.8.4.11/1.8.4.12), are annotated in the PS50^T^ strain genome. At the same time, only the *msr*A gene was annotated in the genome of the type strain *D. formicivorans* Pf12B^T^, which possibly means a lower efficiency of this mechanism compared to the strain PS50^T^. Along with methionine sulfoxide reductase, the TrxA/trxB system, which is composed of thioredoxin (TrxA), thioredoxin reductase (TrxB), and NADPH, plays an important role in the reduction of oxidized proteins. It manages oxidative stress-related damage, acting as a crucial survival factor during oxidative stress (Lu and Holmgren, 2014). The genes of this system are annotated in the genome of strain PS50^T^: the *trx*AB tandem (ACTVJH_04245-04250) and the separately localized *trx*B (ACTVJH_10575). Homologous genes are also annotated in the *D. formicivorans* Pf12B^T^ genome and some *Desulfoplanes* MAGs.

Free metals, particularly iron, are important sources of reactive oxygen species through triggering the Fenton reaction, which can be avoided by storing the metal in specialized proteins. These proteins include bacterioferritin (Bfr), a 24-subunit iron storage protein that has been studied in detail in *Desulfovibrio desulfuricans* ATCC 27774^T^ (Macedo et al., 2003) and *N. vulgaris* Hildenborough (Figueiredo et al., 2012). This protein has been shown to perform two key functions: binding and storing intracellular iron, preventing its accumulation in a free, toxic form; and protecting against oxidative stress by acting, according to the BRENDA portal, as the enzyme ferroxidase (EC: 1.16.3.1), which catalyzes the oxidation of Fe^2+^ to Fe^3+^ coupled to the reduction of O_2_ to form H_2_O. Bacterioferritin synthesis in SRB is encoded by the *bfr* gene, transcription of which, as well as of other oxygen tolerance factors, is controlled by the PerR protein and expression is highly induced upon environmental exposure to oxygen. The PS50^T^ genome contains an annotated *bfr* gene (ACTVJH_08970), whose translated amino acid sequence is 70% homologous to that of the *N. vulgaris* Hildenborough genome. This suggests similar functionality, as well as a preference for using H_2_O_2_ over O_2_ as a co-substrate in Fe^2+^ oxidation (Timóteo et al., 2012). A homologous *bfr* gene has been identified only in the type strain *D. formicivorans* Pf12B^T^, but not in other MAG genomes of *Desulfoplanes*. Thus, both strains, PS50^T^ and Pf12B^T^, appear equipped for oxidative-stress defense, but they differ in the accessory redox systems used for peroxide and thiol-redox homeostasis.

Genes related to compatible-solute metabolism were more prominent in PS50^T^ (Supplementary Table S3). The genome encodes BetA and BetB, suggesting potential conversion of choline to glycine betaine, as well as the glycine betaine transporter OpuD/BetL. PS50^T^ genome also contains trehalose-related annotations, including *ots*B and *tre*T. In contrast, *D. formicivorans* Pf12B^T^ genome contains a betaine/carnitine/choline transporter but lacks the PS50^T^ *bet*AB and *tre*T/*ots*B annotations in this KO table. These differences suggest that PS50^T^ may have a broader capacity for osmotic adjustment, a potentially relevant trait for persistence in saline or redox-fluctuating habitats. This conclusion correlates with a wider range of salinity in the medium at which the PS50^T^ strain grows compared to *D. formicivorans* Pf12B^T^.

#### Hydrogen metabolism

The strain PS50^T^ can support complex hydrogen metabolism thanks to the presence of three genetic clusters in its genome that encode hydrogenases, which differ in the structure of their active sites, intracellular localization, and functional characteristics. The large *ech*ABCDEF cluster (ACTVJH_10880-10905) presumably encodes the multisubunit energy-conserving, membrane-bound Ech-[NiFe]-hydrogenase (ferredoxin) (EC: 1.12.7.2), which catalyzes the reversible reaction of proton reduction to H_2_ formation with a ferredoxin site. This enzyme was originally studied in archaea, where it was shown to contribute to the transmembrane proton motive force and is also involved in energy conservation (Hedderich, 2004; Welte et al., 2010). The presence of an Ech-hydrogenase in SRB was first reported for *Desulfovibrio gigas* DSM 1382^T^, for which the possible role of this hydrogenase as a mechanism for energy conservation was proposed (Rodrigues et al., 2003), and later studied for *(Soli)Desulfovibrio fructosovorans* JJ^T^ (Baffert et al., 2019). According to the BV-BRC portal, the operon of strain PS50^T^ is organized similarly to the operons coding for the Ech hydrogenase from these SRB. The *ech*E gene of the strain PS50^T^, which encodes the large catalytic subunit, are homologous to the *ech*E genes of the JJ^T^ and DSM 1382^T^ strains with 60 and 67% similarity in translated amino acid sequences, respectively, suggesting similar functionality. It is important to note that the genes for Ech-hydrogenase are annotated exclusively in the genome of strain PS50^T^, and have not been identified in the genomes of other *Desulfoplanes*, including the type species *D. formicivorans* Pf12B^T^. Therefore, only for strain PS50^T^ can we suggest the possibility of Ech-hydrogenase participating in the formation of a highly efficient membrane complex with carbon monoxide dehydrogenase (CODH), which directly links CO oxidation to energy conservation by creating a transmembrane proton gradient. As a result of coupling with Ech-hydrogenase, electrons released by CODH are directed into the respiratory chain to support the biological reduction of sulfates. This achieves two advantages for the vital activity of anaerobic SRB: the ability to adapt to substrate-limited environmental conditions and a reduction in the concentration of toxic CO in it (Schoelmerich and Müller, 2020), which is presumably also specific to the strain PS50^T^. The requirement of this enzyme for the hydrogen metabolism of the strain under study may be linked to its aerotolerance, a topic that could be the subject of future research.

Strain PS50^T^ also contains two soluble hydrogenases of the [NiFeSe] and [NiFe] types localized in the periplasm. One of them is presumably the dimeric HysAB-type cytochrome-c3 [NiFeSe] hydrogenase, a selenocysteine-containing enzyme (EC: 1.12.2.1), encoded by the *hys*BA genes (ACTVJH_05790–05795) of the large and small subunits annotated in the genome of strain PS50^T^. *Hys*AB hydrogenase also catalyzes the reversible oxidation of molecular hydrogen into protons and electrons, using cytochrome-c3 as the primary acceptor. When studying this enzyme in the model organism *N. vulgaris* Hildenborough, its interesting catalytic properties were revealed, such as: a strong catalytic bias toward H₂ production with very high activity, resistance to oxygen inactivation, and the ability to increase *hys*BA gene expression during growth in media with low or limiting H₂ concentrations, even to consume minimal amounts of H₂ in the environment (Valente et al., 2005; Caffrey et al., 2007; Zacarias et al., 2018).

Another enzyme is presumably HynAB-type cytochrome-c3 [NiFe] hydrogenase (EC: 1.12.2.1), which is encoded by the genes *hyn*BA (ACTVJH_06100-06105) of the large and small subunits. This enzyme catalyzes a reversible oxidation–reduction reaction of hydrogen using cytochrome c3, depending on the growth conditions: oxidizes hydrogen in the presence of sulfate and produces some hydrogen during fermentation of pyruvate or lactate without electron acceptor(s). It is predominantly found in sulfate-reducing bacteria, and in some of them it is the single hydrogenase of the [NiFe] type (Hatchikian et al., 1995).

In addition, the PS50^T^ genome contains auxiliary *hyp*AB genes encoding [NiFe]-hydrogenase nickel incorporation proteins, *hyp*CDE genes encoding [NiFe]-hydrogenase metallocentre assembly proteins, and *hya*D genes encoding [NiFe]-hydrogenase maturation proteins.

It should be noted that the PS50^T^ genome contains only [Ni-Fe]-hydrogenase genes, which are recognized as being more tolerant to the toxic effects of oxygen on metal-containing enzyme active sites than [Fe-Fe]-variants (Bingham et al., 2012; Jespersen et al., 2025). This does not contradict the experimentally demonstrated increased level of aerotolerance for this SRB.

#### Cofactor metabolism, transport, and cellular interaction

Cobalamin and corrinoid-related KOs were enriched in PS50^T^ genome, including several *cob*/*cbi* genes and the cobalamin transporter components BtuC and BtuD. *D. formicivorans* Pf12B^T^ genome retained a smaller corrinoid-associated repertoire. This pattern suggests greater flexibility in corrinoid biosynthesis, remodeling or uptake by PS50^T^, which may be relevant in environments where vitamin B12 availability varies (Supplementary Table S3).

PS50^T^ also encodes a phosphonate ABC transport system, molybdate transport components, a P-type copper transporter and additional secretion/adhesion-associated genes, including LapC and LapE. These features suggest an expanded capacity for nutrient acquisition, metal/cofactor handling and surface-associated growth relative to *D. formicivorans*. Conversely, *D. formicivorans* Pf12B^T^ contains functions not detected in strain PS50^T^, including ethanolamine ammonia-lyase, ferritin formation, iron-siderophore transport and a spermidine/putrescine ABC transporter.

#### Defense systems, motility and genus-level conserved traits

Defense and persistence-related annotations were more numerous in PS50^T^. The genome contains multiple genes coding toxin-antitoxin module components, including HipA-family kinases and VapBC, YafQ-YefM, YoeB and StbD-related annotations (Supplementary Table S3). PS50^T^ genome also carries additional type III-B Cmr/Cas components. *D. formicivorans* Pf12B^T^ genome is not devoid of defense genes, as it encodes a *cas*/*csd* CRISPR-associated gene set and some toxin-related annotations, but the PS50^T^ repertoire is broader.

Both strains encode flagellar and chemotaxis functions. PS50^T^ has a larger repertoire of methyl-accepting chemotaxis proteins (15 versus 8), suggesting a broader capacity to sense chemical gradients. Both genomes also contain *lar*BCE, encoding the nickel-dependent lactate racemase maturation system, indicating that lactate interconversion is a conserved feature within the compared *Desulfoplanes* genomes.

Therefore, the strain PS50^T^ is adapted to the temperature and salinity of its habitat, is able to grow in the presence of 5% oxygen and survive when reservoir and injected water mix, spreading through the reservoir and in the water treatment system. The ability to grow in the absence of an energy source (hydrogen or organic substrates), receiving electrons from ferrous iron (Fe^2+^), determines its participation in the processes of corrosion of oil equipment.

## Conclusion

In this work, a new sulfate-reducing bacterium, strain PS50^T^, has been isolated from a petroleum reservoir. The strain caused corrosion of steel coupons. Its genome contains the main genes, *sat* and *dsr* genes of sulfate reduction, *his*, *hyn*, and *echo* genes of hydrogenase and *feo* genes of iron uptake, which confirms the strain’s ability to participate in the processes of steel biocorrosion. A phylogenetic analysis of the 16S rRNA gene and 120 universal marker genes has shown the strain to belong to a new species of the genus *Desulfoplanes*. The KEGG comparison shows that PS50^T^ genome shares the sulfate-reducing core and major anaerobic metabolic framework of *D. formicivorans* Pf12B^T^, supporting its assignment to the genus *Desulfoplanes*. The strain PS50^T^ differs from *D. formicivorans* Pf12B^T^ by a wider salinity range for growth, inability to grow at high temperature (50 °C), glucose fermentation, and cellular fatty acid composition, as well as by a larger genome, more strain-specific KOs and accessory functions associated with osmoprotection, redox stress response, corrinoid metabolism, transport, chemotaxis, surface interaction and defense. These genomic distinctions provide functional support for recognizing PS50^T^ as a separate species, while leaving room for phenotypic experiments to define which predicted capacities are expressed under laboratory conditions. Thus, phenotypic characteristics and phylogenetic and genomic analyses described above indicate that strain PS50^T^ represents a new species of the genus *Desulfoplanes*, for which the name *Desulfoplanes aerotolerans* sp. nov. is proposed. The description of the species *Desulfoplanes aerotolerans* sp. nov. is given in the protologue (Table 3).

**Table 3:** Protologue description of *Desulfoplanes aerotolerans* sp. nov.

Parameter	*Desulfoplanes aerotolerans* sp. nov.
Genus name	Desulfoplanes
Species name	*Desulfoplanes aerotolerans*
Species status	sp. nov.
Species etymology	a.e.ro.to’le.rans. Gr. masc. n. aêr, air; L. pres. part. tolerans, tolerating; N.L. masc. Part. adj. Aerotolerans, air-tolerating, referring to the oxygen tolerance of the organism
Designation of the Type Strain	PS50^T^
Strain Collection Numbers	VKM B-4014^T^ = KCTC 35101^T^
Genome accession number	GCF_053851535.1
Genome status	Complete
Genome size	3.5 Mb
GC mol%	53.5
16S rRNA gene accession nr.	OQ348127.2
Description of the new taxon and diagnostic traits	The cells are Gram-stain negative, non-spore-forming, motile, slightly curved rods or vibrions. Growth is observed in the presence of 1.0–10.0% (w/v) NaCl (optimum, 4.0–5.0% NaCl), at pH 5.6–8.6 (optimum, pH 7.5), and at 15–37 °C (optimum, 25–30 °C) under sulfate-reducing conditions. Grow both anaerobically and aerobically in the presence of 5% (v/v) atmospheric O_2_. In anaerobic conditions reduces sulfate to sulfide in media with formate, lactate, and yeast extract as carbon and energy sources, but does not use acetate, pyruvate, propionate, malate, fumarate, succinate, methanol, ethanol, glucose, and benzoate. Hydrogen is utilized as an electron donor for sulfate reduction in the presence of acetate as a carbon source. Lactate is oxidized with the production of acetate. Fermentative growth is observed with pyruvate, lactate, fumarate, and glucose. It uses sulfate, sulfite, thiosulfate, and elemental sulfur as electron acceptors in the presence of lactate, but does not use nitrate. The predominant cellular fatty acids are C_17:1_ ω9, iso-C_15:0_, and anteiso-C_15:0_. The major polar lipids include phosphatidylethanolamine, phosphatidylglycerol, and diphosphatidylglycerol. The genome size of the type strain is 3.5 Mb with a genomic G + C content of 53.5 mol%. The type strain, PS50^T^ (VKM B-4014^T^ = KCTC 35101^T^), was isolated from the Arkhangelskoe oil field, Tatarstan, Russian Federation. The GenBank/EMBL/DDBJ accession number for the 16S rRNA gene sequence is OQ348127.2 and the genomic assembly accession number is GCF_053851535.1 = JBSPZA000000000.1.
Country and region of origin	Russian Federation, Tatarstan
Date of isolation	January 2023
Source of isolation	Production water from the Arkhangelskoe oil field
Sampling date	June 2021
Latitude, Longitude	55°13′10.6″N, 51°22′33.9″E
Depth (meters below sea level)	988–998
Number of strains in study	1
Information related to the Nagoya protocol	Not applicable

## Data Availability

The 16S rRNA gene sequence and the genomic assembly of strain PS50T have been deposited at DDBJ/EMBL/GenBank under the accession numbers OQ348127.2 and GCF_053851535.1 (=JBSPZA000000000.1), respectively.
