# Supplementary material for: Phenotypic and genomic characterization of a halophilic sulfate-reducing bacterium Desulfoplanes aerotolerans sp. nov. from a petroleum reservoir and its possible implication in pipeline corrosion
Source: Front Microbiol. 2026 Oct 14;17:1968038. doi: 10.3389/fmicb.2026.1968038 (PMC13713477; doi:10.3389/fmicb.2026.1968038)
Supplement: Supplementary file 1 [file Data_Sheet_1.PDF]

Supplementary Material

Phenotypic and Genomic Characterization of a Halophilic Sulfate-Reducing Bacterium *Desulfoplanes aerotolerans* sp. nov. from a Petroleum Reservoir and Its Possible Implication in Pipeline Corrosion

Salimat K. Bidzhieva[1], Tatyana P. Tourova[1], Denis S. Grouzdev[2], Salima R. Soloveva[1], Diyana S. Sokolova[1], Vera M. Tereshina[1], Andrey B. Poltaraus[3], Andrey V. Mardanov[4] and Tamara N. Nazina[1*]

[1]Winogradsky Institute of Microbiology, Research Center of Biotechnology, Russian Academy of Sciences, Moscow, Russia

[2] School of Marine and Atmospheric Sciences, Stony Brook University, Stony Brook, NY, USA

[3]Engelhardt Institute of Molecular Biology, Russian Academy of Sciences, Moscow, Russia

[4]Institute of Bioengineering, Research Center of Biotechnology, Russian Academy of Sciences, Moscow, Russia

***Corresponding Author**
Tamara N. Nazina
Email: nazina@inmi.ru
ORCID: 0000-0002-6586-2467

This file includes:

Methods for determining the corrosion potential and biocide resistance of the strain $PS50^T$

Supplementary Figures S1 to S11

Supplementary Tables S1 to S3

Methods for determining the corrosion potential and biocide resistance of the strain PS50^T

The corrosion potential of the strain PS50^T was evaluated on SMM medium with a steel coupon (Steel-20) under conditions of mixotrophic (SO_4^{2-}, CO_2 and acetate) and heterotrophic (SO_4^{2-} and lactate) growth at 30 °C. In the case of mixotrophic growth, no H_2 was introduced into the gas phase to assess the ability of the strains to use coupon iron as an electron donor. The gas phase was represented by carbon dioxide. A 10-day culture was added to the test tubes. Incubation was carried out at 30 °C. The concentration of the formed sulfide was determined by 2, 5, 7, 9, 15, 20, 30, 40, 60 and the 120th day. Abiogenic corrosion was controlled by a steel coupon incubated in a sterile environment.

The corrosion rate was determined by gravimetric method (GOST ISO 9226-2022) using control and experimental coupons for 20, 60 and 120 days of incubation. First, the steel coupon was mechanically cleaned from corrosive deposits, then it was immersed for 30 seconds in a solution of concentrated hydrochloric acid, washed with water, immersed in a saturated soda solution for 10 seconds to neutralize acid residues, washed in distilled water and dried. Next, the samples were weighed, and the corrosion rate was calculated using the formula (mm/yr):

$$\text{Corrosion Rate} = \frac{(m1-m2)\times 24\times 365}{S\times T\times \rho}, \qquad \text{Equation 1}$$

where m1 is the mass of the coupon before the tests, g; m2 is the mass of the coupon after the tests, g; S is the surface area of the coupon, mm^2; T is the duration of the tests, h; 24×365 is the conversion factor of hours per year; ρ is the density of the coupon material (7.82×10^{-3} g/mm^3).

To determine the resistance of the strain PS50^T to biocides, 3 commercial biocides were used, designated as A, B, and C, containing polyhexamethylene guanidine hydrochloride, formaldehyde, and glutaraldehyde, respectively, as the active base. The concentration of the bactericidal base in the reagents is not specified by the manufacturer. Biocides B and C are recommended for inhibiting the growth of sulfate-reducing bacteria in the petroleum industry. Biocide A is recommended by the manufacturer to combat fungal and bacterial agents in various industries, and even for domestic use. The effectiveness of different biocide dosages was compared on a 4-day culture of the strain PS50^T obtained on SMM medium with lactate and sulfate. An aliquot culture (106 cells in 1 ml) was added to sterile solutions of SMM medium without a substrate containing 40, 60, 80, or 100 mg of biocide in 1 liter and kept for 24 hours. After exposure to the biocide, the culture was transferred to a sterile SMM medium with lactate and sulfate and incubated for 14 days at 30 °C. Cell survival was assessed by the formation of sulfide in the medium.

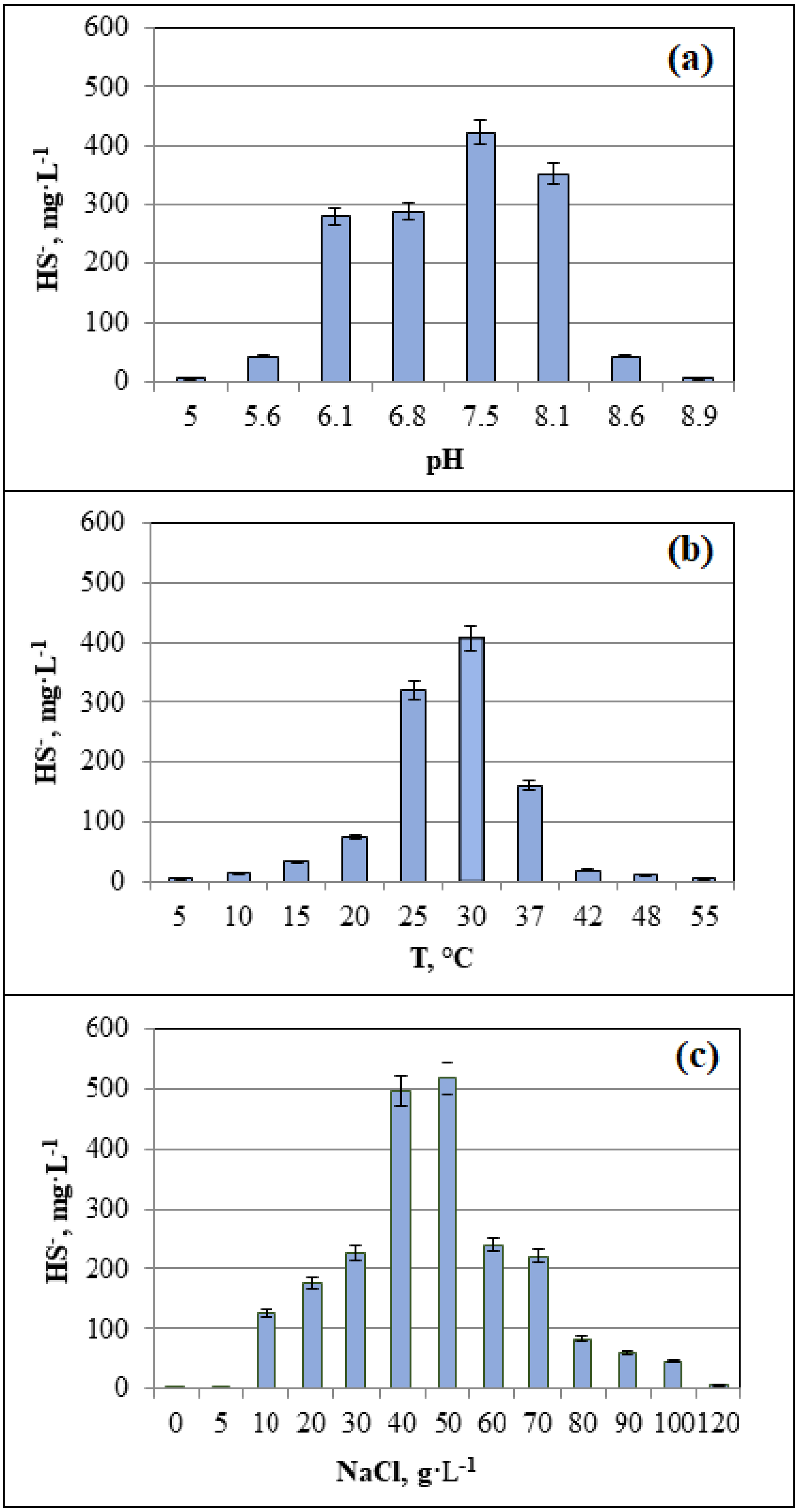

Supplementary Figure S1. Growth profiles of the strain PS50^T at various pH (a), temperatures (b), and NaCl concentrations (*w/v*, %) (c) after 10 days of incubation.

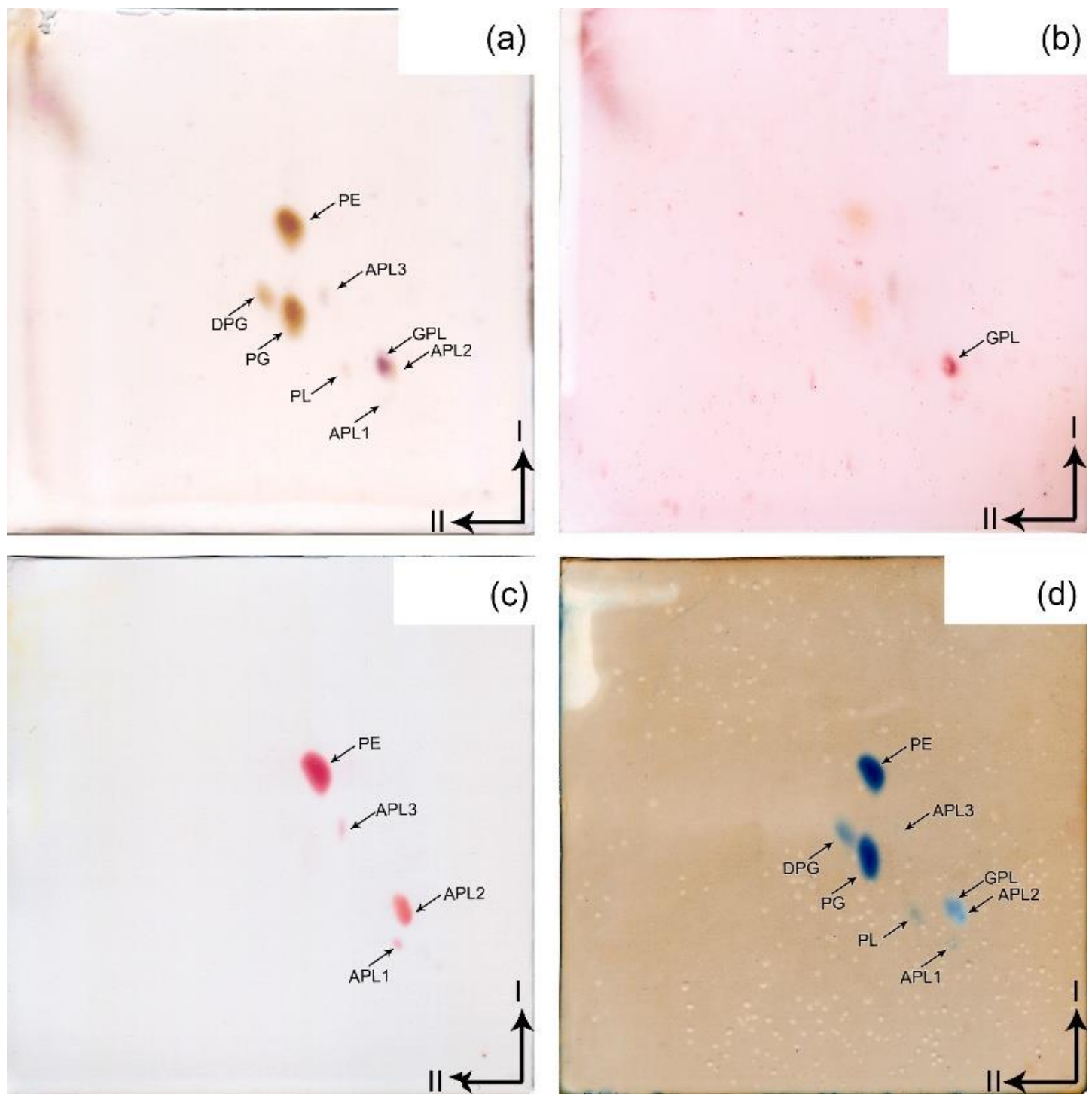

Supplementary Figure S2. Chromatograms of polar lipids from the strain PS50^{T}. 100–125 µg of lipids was applied to the plate. The components on the two-dimensional thin layer chromatograms were visualized by staining (a) with 5% sulfuric acid in ethanol and heating at 180°C for 15 min; (b) thymol; (c) ninhydrin; and (d) molybdenum blue (Vaskovsky modification of Dittmer-Lester reagent). Abbreviations: APL, aminophospholipids; DPG, diphosphatidylglycerol; GPL, glycophospholipids; PE, phosphatidylethanolamine; PG, phosphatidylglycerol; PL, phospholipid. The solvent systems used were chloroform-methanol-water (65:25:4, *v/v*) for the first dimension (I) and chloroform-acetone-methanol-acetic acid-water (50:20:10:10:5, *v/v*) for the second dimension (II).

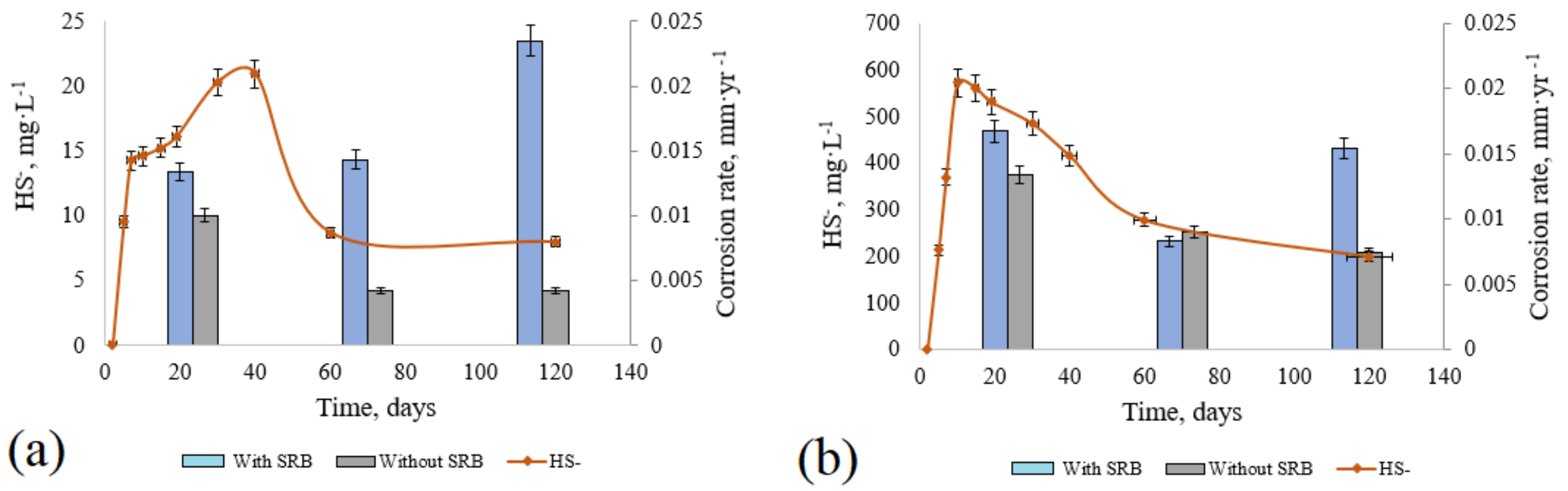

Supplementary Figure S3. The formation of sulfide by the strain PS50^{T} under mixotrophic (a) and heterotrophic (b) conditions and the corrosion rate of the steel coupon. The strain was incubated for 120 days at 30 °C.

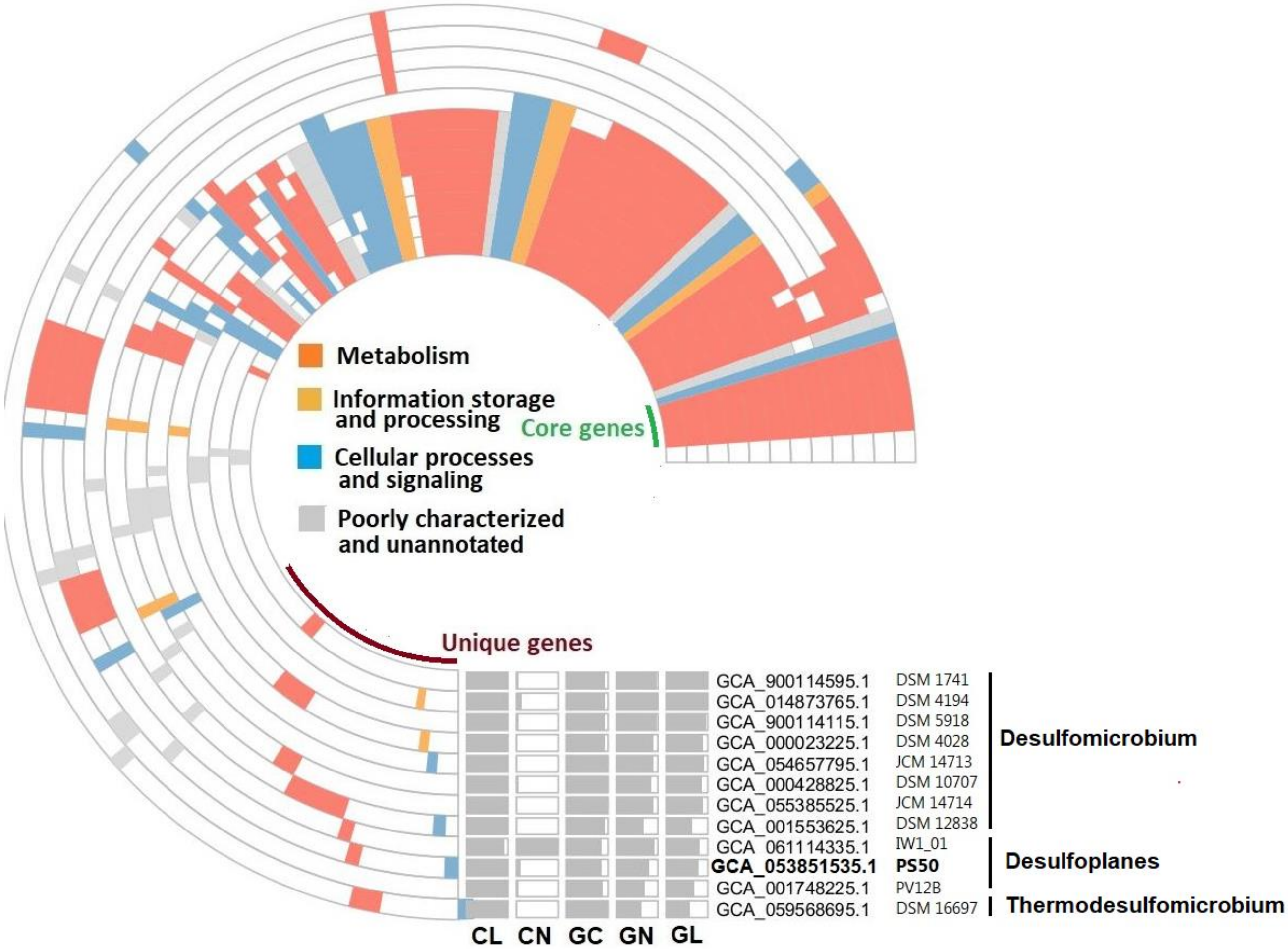

Supplementary Figure S4. The pan-genome profile of SRB of the *Desulfomicrobiaceae* family including all type strains in genera *Desulfomicrobium* and *Thermodesulfomicrobium*, as well as the type strains and MAGs in genus *Desulfoplanes*. Primary COG annotation shows the core genes and unique genes. CL, CN, GC, GN, and GL represent genome length, contig number, GC content, gene number, and genome completeness, respectively. The AC number of the studied strain PS50^T is marked by boldface.

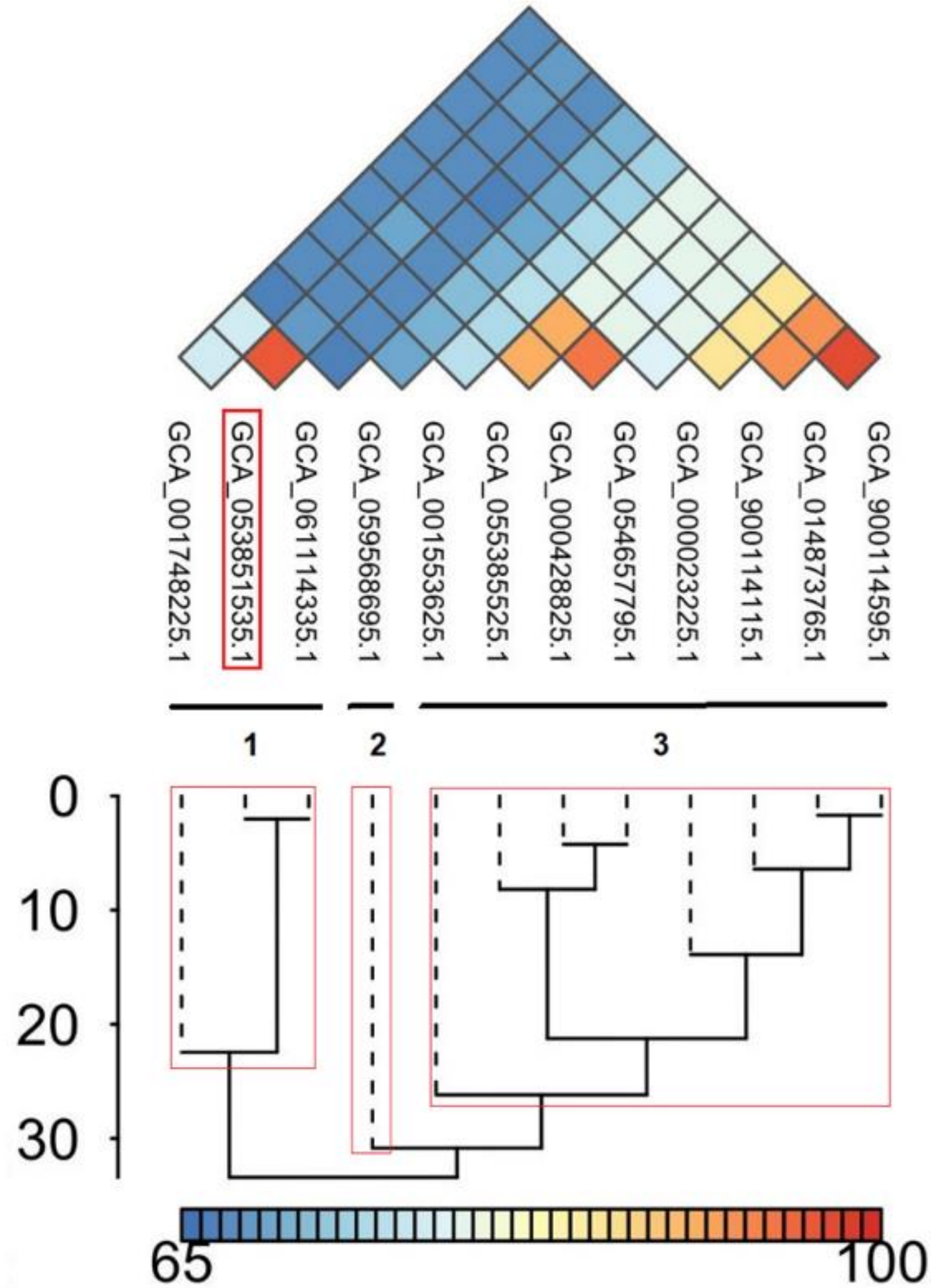

Supplementary Figure S5. ANI matrix (%) of the strain PS50^T genome and other genomes of *Desulfomicrobiaceae* family including genera *Desulfoplanes* (1), *Thermodesulfomicrobium* (2), and *Desulfomicrobium* (3) and their cladogram. The AC number of the studied strain PS50^T is marked by red frame.

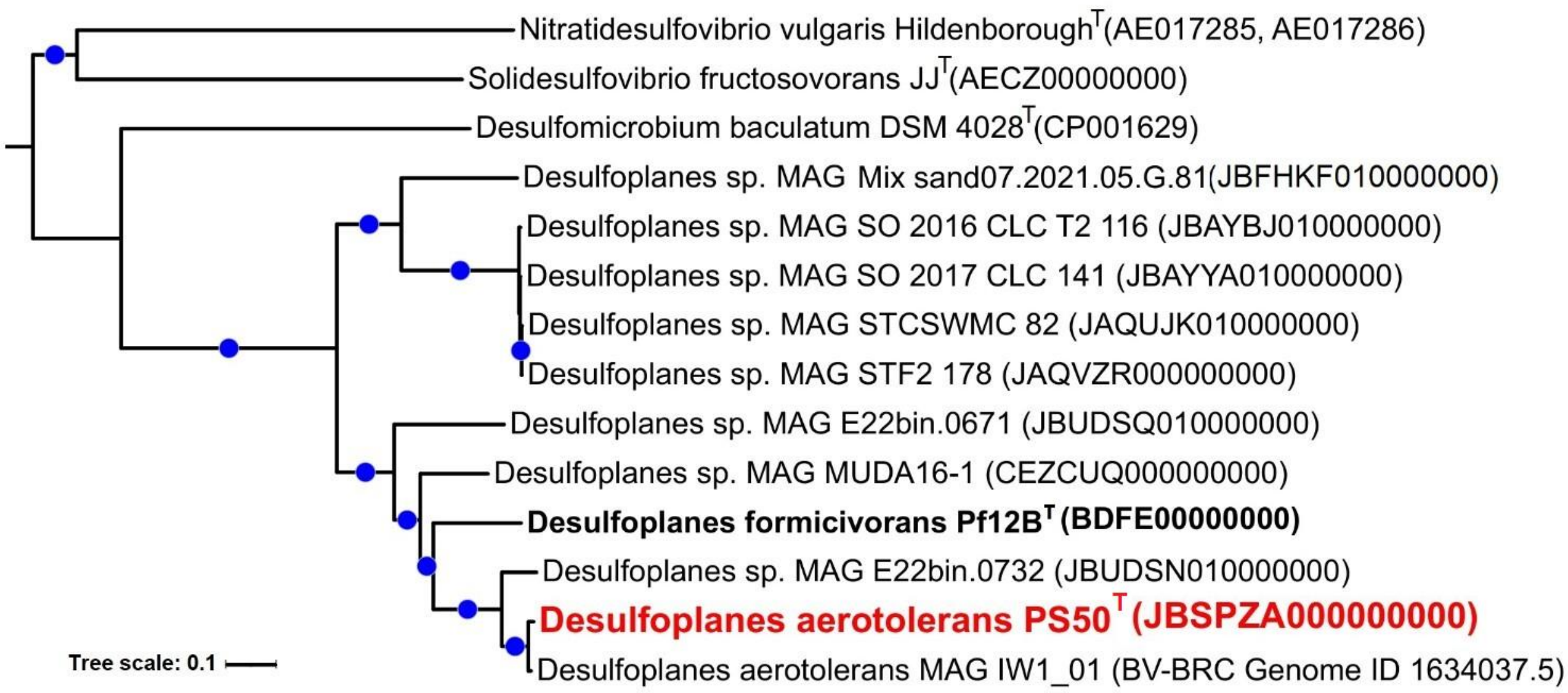

Supplementary Figure S6. The maximum-likelihood phylogenetic tree derived from 67 single-copy proteins shows the position of strain PS50^T within the genus *Desulfoplanes*. Bar: 0.1 amino acid substitutions per site. Bootstrap values >90 are shown as blue circles at the branching points. The tree was rooted using midpoint. Accession numbers for the GenBank AC numbers are indicated in brackets. The name of the studied strain PS50^T is marked by red boldface. The name of the type species of *Desulfoplanes* genus is marked by boldface.

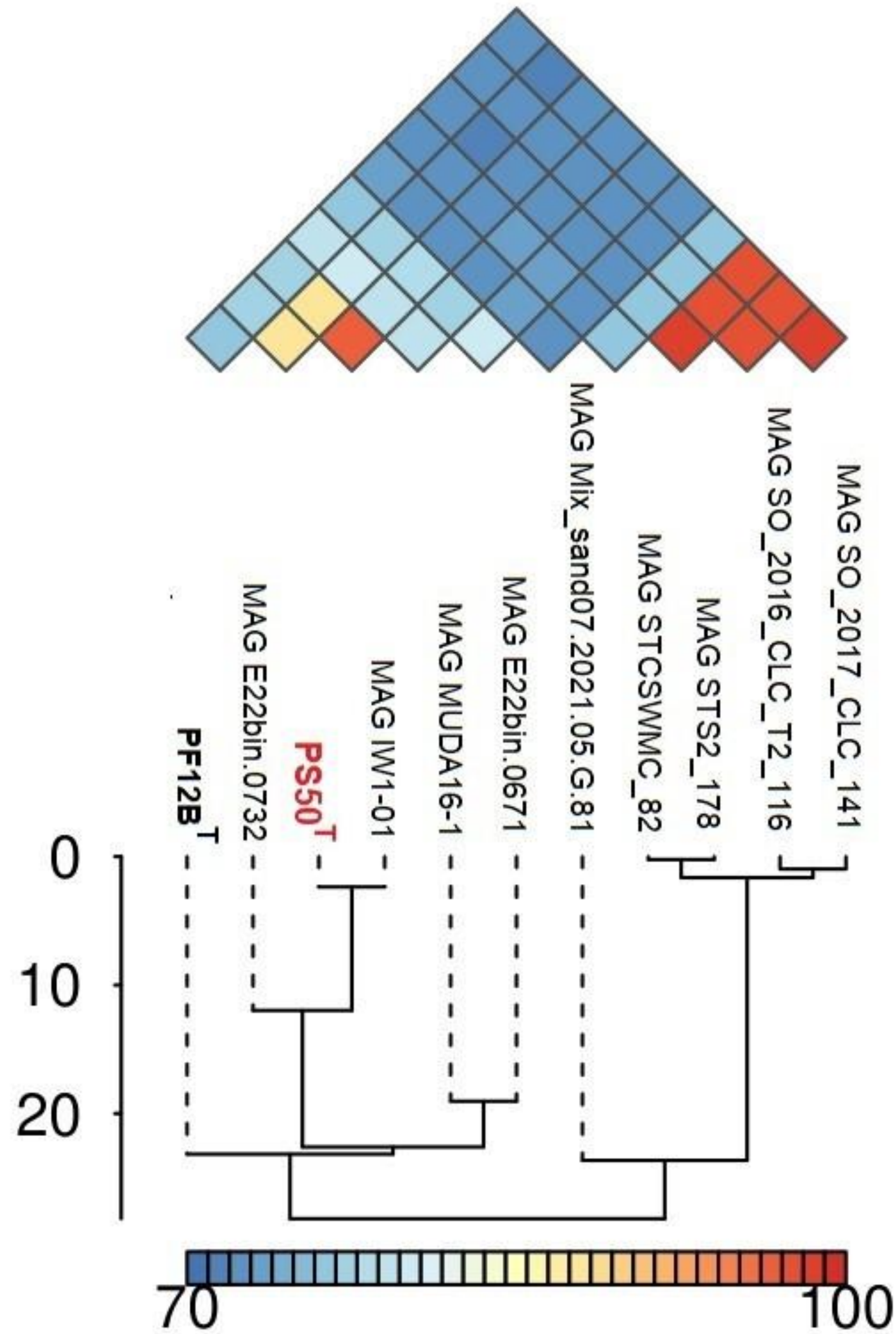

Supplementary Figure S7. ANI matrix (%) of the strain PS50^{T} genome and other genomes of *Desulfoplanes* genus and their cladogram. The name of the studied strain PS50^{T} is marked by red boldface. The name of the type species of *Desulfoplanes* genus is marked by boldface.

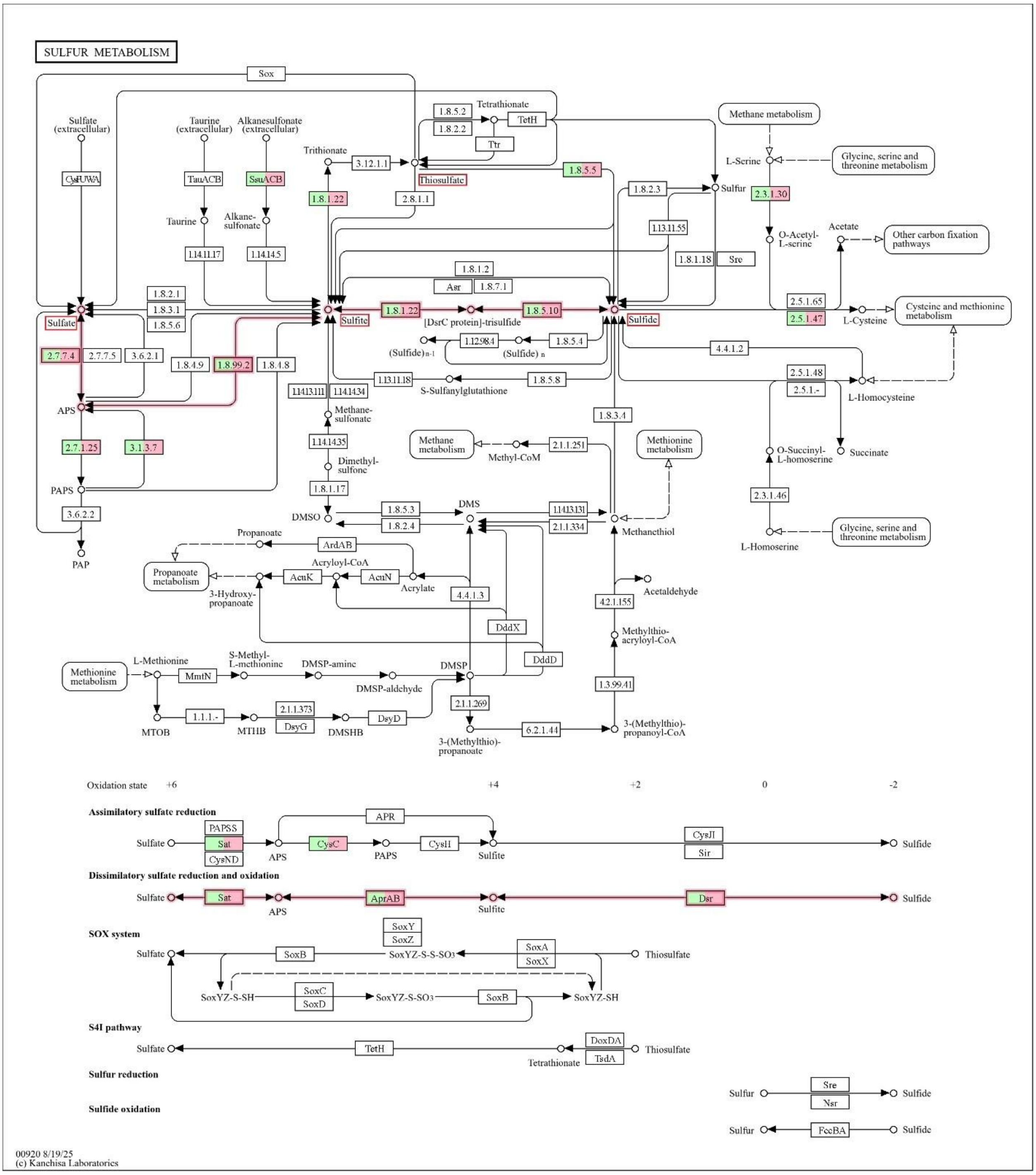

Supplementary Figure S8. Comparative KEGG map (00920) of sulfur metabolism based on the genome analysis of strains PS50^{T} and *D. formicivorans* Pf12B^{T}. The enzymes encoded by the genes annotated in the genome are highlighted in green for the strain PS50^{T} and in red for *D. formicivorans* Pf12B^{T}. The presumptive modules of dissimilatory sulfate reduction are highlighted in pink.

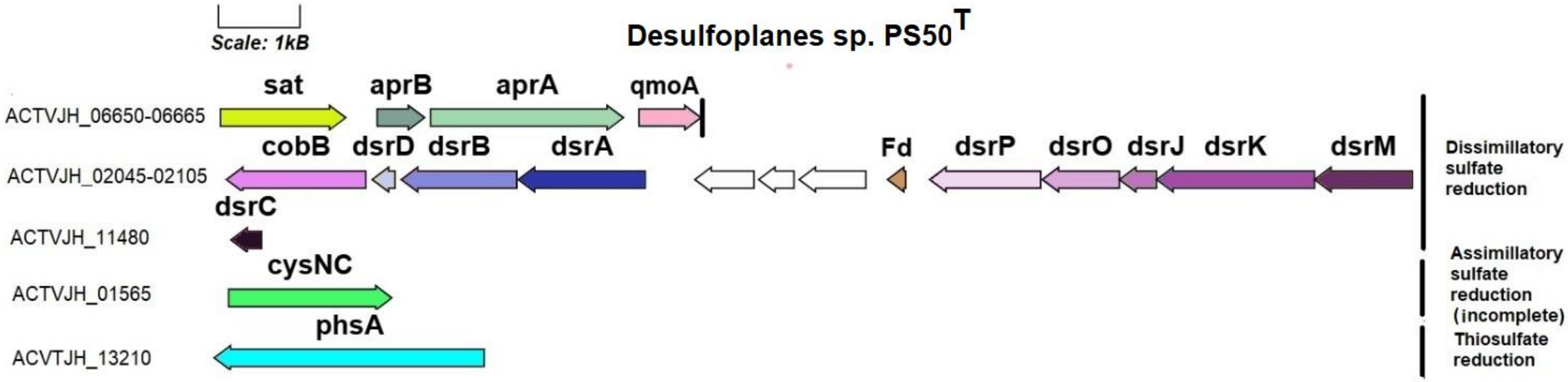

Supplementary Figure S9. The genes presumably encoding sulfur metabolism in the genome of the strain PS50^T. Abbreviations: *sat*, sulfate adenylyltransferase; *apr*AB, adenylylsulfate reductase; *qmo*A, protein of adenylsulfate reductase-associated electron transfer complex; *dsr*ABD, dissimilatory sulfite reductase; *dsr*MKJOP, [DsrC]-trisulfide reductase; *dsr*C, clustered with dissimilatory sulfite reductase; *cys*NC, bifunctional adenylyl-sulfate kinase; *phs*A*(psr*A), thiosulfate reductase / polysulfide reductase. Scale bar, 1000 bp.

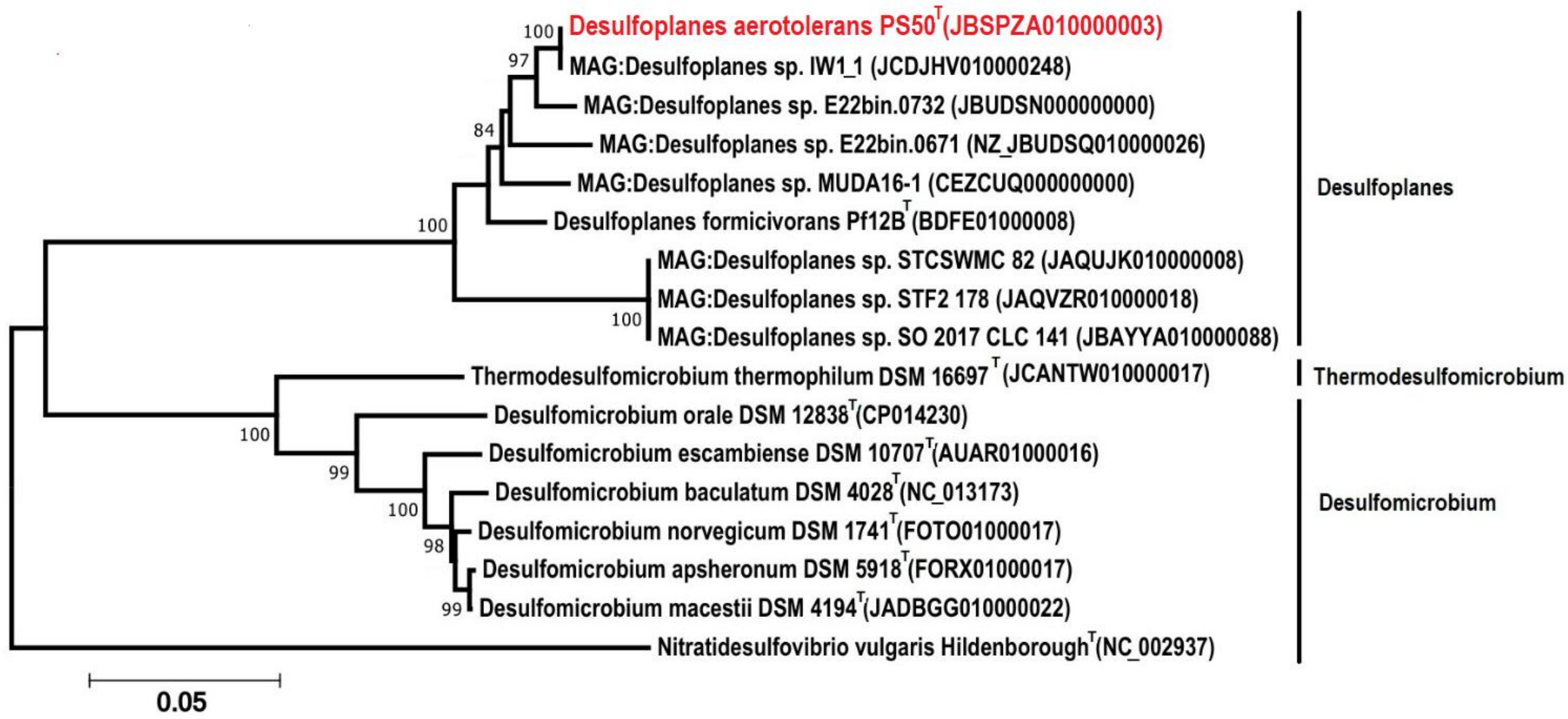

Supplementary Figure S10. The maximum-likelihood tree based on the translated amino acid sequences of the *dsr*AB genes showing the phylogenetic position of strain PS50^T within the family *Desulfomicrobiaceae*. Bar: 0.05 amino acid substitutions per site. Bootstrap values from 1000 trees >80% are shown at the branching points. The tree was rooted using *Nitratidesulfovibrio vulgaris* Hildenborough. Accession numbers for the GenBank are indicated in brackets. The name of the studied strain PS50^T is marked by red.

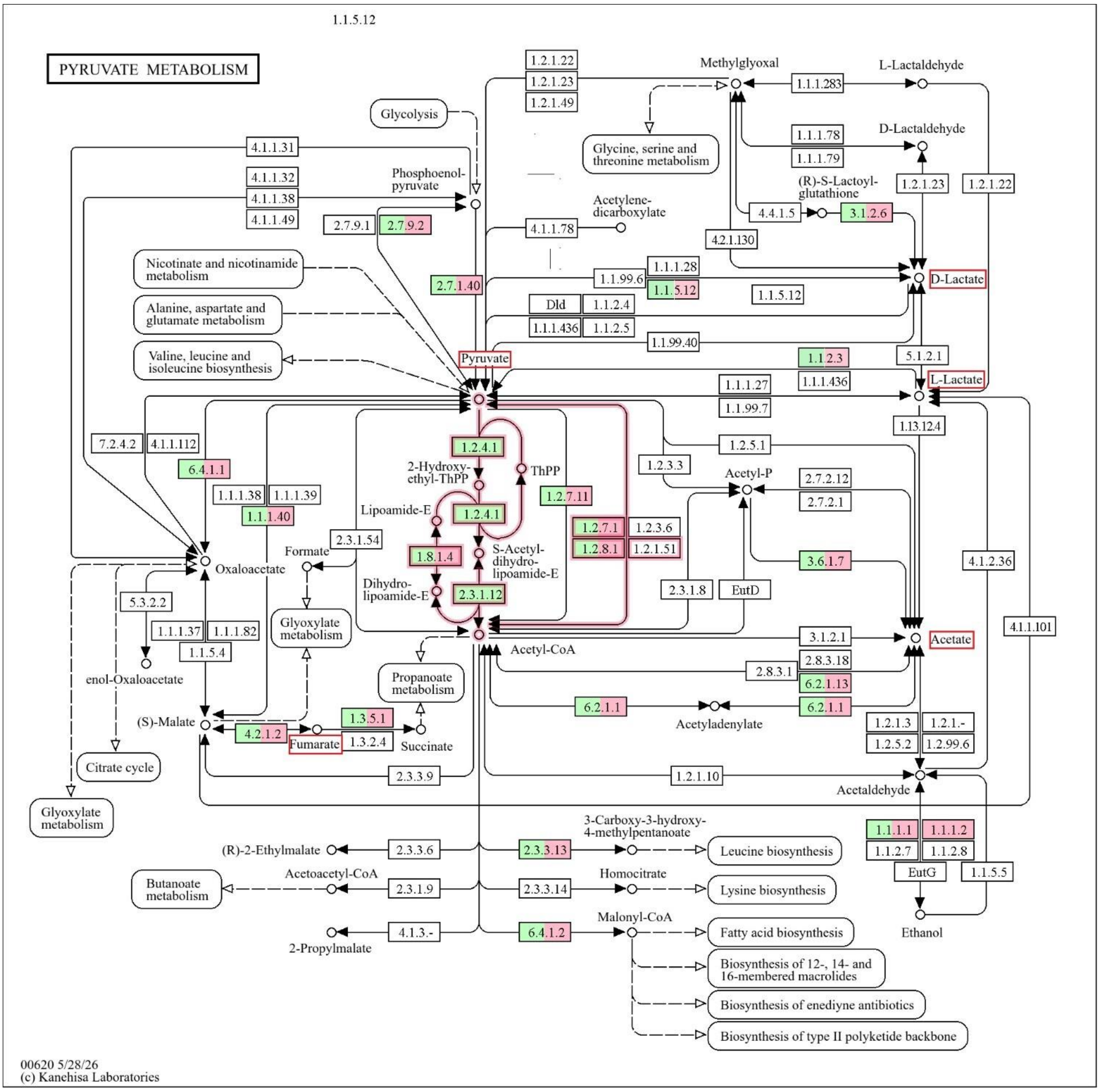

Supplementary Figure S11. Comparative KEGG map (00620) of pyruvate metabolism based on the genome analysis of strains PS50^{T} and *D. formicivorans* Pf12B^{T}. The enzymes encoded by the genes annotated in the genome are highlighted in green for the strain PS50^{T} and in red for *D. formicivorans* Pf12B^{T}. The presumptive pyruvate oxidation modules are highlighted in pink.

Supplementary Table S1. Cellular fatty acid composition* (% of the total) of strains PS50^T and *Desulfoplanes formicivorans* Pf12B^T.

Fatty acid	Strain	
	**PS50^T**	**Pf12B^T**
$C_{12:0}$ 2-OH		0.2
iso-$C_{14:0}$		1.0
$C_{14:0}$		2.5
iso-$C_{15:1}$ F		0.2
iso-$C_{15:0}$	**26.74**	**6.0**
anteiso-$C_{15:0}$	**16.83**	4.7
iso-$C_{16:0}$	2.25	4.3
$C_{16:1}$ c9		0.3
$C_{16:0}$	3.02	**41.1**
iso-$C_{15:0}$ 3-OH		0.9
$C_{15:0}$ 2-OH		0.4
$C_{17:1}$ ω9	**34.20**	
anteiso-$C_{17:1}$ c9		0.2
$C_{17:1}$ ω10	1.10	
iso-$C_{17:0}$	5.89	0.8
anteiso-$C_{17:0}$	7.45	1.7
$C_{17:0}$ cyclo		0.8
$C_{17:0}$		1.4
$C_{16:0}$ 3-OH		1.9
$C_{18:1}$ c9		1.5
$C_{18:1}$ ω9	1.71	
$C_{18:0}$	0.81	**20.6**
$C_{19:0}$ cyclo c8		1.9
$C_{18:0}$ 3-OH		1.1

*Data of this study for strain PS50^T; data for *D. formicivorans* Pf12B^T were obtained from Watanabe et al. (2015). Dominant fatty acids are indicated in bold.

Supplementary Table S2. The effect of biocides in different concentrations on the formation of sulfide by the strain PS50^T.

**Biocide**	**The composition of the biocide**	**Biocide concentration, mg/L**	HS^-, mg/L
A	Glutaraldehyde, oxalic aldehyde, methanol	40	411
		60	493
		80	430
		100	416
B	Formaldehyde (5-7 wt. %), methanol (30-35 wt. %), heterocyclic polymer (10-15%)	40	451
		60	482
		80	448
		100	380
C	Polyhexamethylene guanidine hydrochloride	40	0
		60	0
		80	0
		100	0

Supplementary Table S3. Functional differences between genomes of *Desulfoplanes aerotolerans* PS50^T and *D. formicivorans* Pf12B^T with KO identifiers used for interpretation.

Functional block	KO / genes used for comparison	PS50^T	Pf12B^T	Main difference / interpretation
1	2	3	4	5
Genome-level KO summary	Total proteins; KO-assigned proteins; strain-specific KOs	3,078 proteins; 1,559 KO-assigned; 165 PS50-specific KOs	2,657 proteins; 1,414 KO-assigned; 96 Pf12B-specific KOs	PS50^T has a larger gene complement and a larger accessory KO set.
Dissimilatory sulfate reduction core	sat K00958; aprA K00394; aprB K00395; dsrA K11180; dsrB K11181; dsrC K23077; dsrMKJOP K27187-K27191	Present; sat x2; canonical sulfate-reduction core retained	Present; sat x2; canonical sulfate-reduction core retained	Core sulfate-reducing metabolism is conserved in both genomes.
Qmo electron-transfer module	qmoA K16885; qmoB K16886; qmoC K16887	qmoA detected as two primary KO annotations; qmoB/C present	qmoB/C present; qmoA not detected as primary KO annotation	Difference in qmoA annotation suggests variation in the APS-reductase electron-transfer module.
Pyruvate oxidation	pdhA K00161; pdhB K00162; pdhC/aceF K00627; pdhD/lpdA K00382	Primary pdhA/pdhB/pdhC annotations detected; pdhD shared	No primary pdhABC assignments; pdhD shared; weak/secondary related hits present	PS50^T has stronger annotation support for PDH-related pyruvate oxidation under redox-variable conditions.
Anaerobic pyruvate metabolism	por/nifJ K03737; pflA/pflC/pflE K04069	por/nifJ present; PFL-activating enzyme present	por/nifJ present; PFL-activating enzyme present	Anaerobic pyruvate metabolism is conserved in both strains.
CO and formate metabolism	cooS K00198; cooF K00196; fdhA K00123; fdhB K00124; fhs K01938	More CODH annotations; cooF detected; fewer fdhA/fdhB annotations	Fewer CODH annotations; cooF not detected; more fdhA/fdhB plus fhs	PS50^T is more CO/CODH-skewed, whereas Pf12B^T is more formate-skewed.
Respiratory electron transport	Nuo/Complex I: nuoA-N K00330-K00343; cydA K00425; cydB K00426	Broader primary Nuo annotation set; cydAB present	Several Nuo subunits present; fewer primary Nuo assignments; cydAB present	Both encode cytochrome bd oxidase; PS50^T has a broader annotated Nuo repertoire.

Table S3. Cont.

1	2	3	4	5
Oxidative stress defense	katE K03781; sodB K04564; gor K00383; ahpC K24119/K03386; tpx K11065; ahpE K24120	katE, sodB, cydAB; gor; NADH-dependent AhpC	katE, sodB, cydAB; PRDX2/4-like AhpC, Tpx and AhpE	Both have oxygen/ROS defenses, but accessory peroxide/redox systems differ. Accessory redox systems differ between the strains.
Compatible solutes / osmoprotection	betA K00108; betB K00130; opuD/betL K05020; otsB K01087; treT K13057; BCCT K03451	betAB, OpuD/BetL, otsB and treT detected	BCCT transporter detected; betAB and treT/otsB not detected in primary KO table	PS50^T has stronger annotation support for betaine synthesis/transport and trehalose-related osmoprotection.
Cobalamin / corrinoid functions	cobL/cbiET K00595; cobI/cbiL K03394; cobJ/cbiH K05934; cobM/cbiF K05936; cbiD K02188; btuC/D K25027/K25028; metE K00549	Enriched cob/cbi repertoire; btuCD present; metE present	Reduced corrinoid-associated repertoire in this KO table	PS50^T appears more flexible in corrinoid biosynthesis/remodeling or uptake.
Nutrient and metal transport	phnCDE K02041/K02042/K02044; modA/B K02020/K02018; copB K01533; znuABC K09815-K09817; arsB/arsC K03325/K03741	Phosphonate, molybdate, copper, zinc and arsenic-related systems detected	Some metal/ion systems detected; phosphate/iron/polyamine features more evident	PS50^T has additional nutrient/cofactor and metal-homeostasis functions, but Pf12B^T has its own distinct transport traits.
Surface interaction / secretion	lapC K12542; lapE K12543; gspJ K02459; polysaccharide/export genes wza K01991, exoP K16554, epsF K25590	LapC/LapE and several surface/export-related annotations detected	LapC/LapE not detected in KO table	Supports possible differences in attachment, envelope remodeling or biofilm-associated behavior.
Defense and persistence	HipA K07154; VapBC K18828/K18829; YafQ/YefM K19157/K19159; YoeB K19158; StbD K18923; Cmr/Cas K19076/K09000/K19142; Cas/Csd genes	Expanded TA annotations; additional type III-B Cmr/Cas components plus cas/csd genes	cas/csd CRISPR genes present; limited TA-related annotations	PS50^T has broader defense/persistence repertoire; Pf12B^T is not defense-free.
Motility and chemotaxis	mcp K03406; cheA K03407; cheW K03408; cheB K03412; cheD	15 MCP annotations; additional chemotaxis-associated functions	8 MCP annotations; core motility/chemotaxis present	PS50^T may sense a broader range of chemical gradients.

Table S3. Cont.

1	2	3	4	5
	K03411; cheZ K03414; motA/B K02556/K02557			
Conserved lactate racemase system	larB K06898; larC K09121; larE K06864	Present	Present	Shared genus-level trait; not a differentiating feature.
Functions more evident in Pf12B^T	eutB/C K03735/K03736; ftnA K02217; potABCD K11069-K11072; siderophore/iron transport K02014/K23188	Not detected as primary KO annotations	Detected	Useful comparator traits: ethanolamine use, iron storage/uptake and polyamine transport are stronger in Pf12B^T.

Abbreviations: KO, KEGG Orthology; MCP, methyl-accepting chemotaxis protein. KO presence indicates detection in the comparative annotation table. Note: functional assignments are based on KEGG Orthology annotation and are interpreted in the context of comparative genome analysis.
